# Incidence and mortality of nonmelanoma skin cancer in Europe: current trends and challenges

**DOI:** 10.1007/s12094-025-03985-z

**Published:** 2025-07-11

**Authors:** Mercedes Sendín-Martín, Rocío C. Bueno-Molina, Juan-Carlos Hernández-Rodríguez, Lucía Cayuela, Aurelio Cayuela, José-Juan Pereyra-Rodríguez

**Affiliations:** 1 Department of Dermatology, Virgen del Rocío University Hospital, Seville, Spain; 2 Department of Internal Medicine, Hospital Severo Ochoa, Leganés, Spain; 3 Unit of Public Health, Prevention and Health Promotion, South Seville Health Management Area, Seville, Spain; 4 Department of Medicine, University of Seville, Seville, Spain

**Keywords:** Non melanoma skin cancer, Basal cell carcinoma, Squamous cell carcinoma, Incidence, Mortality, Europe

## Abstract

**Purpose:**

Nonmelanoma skin cancer (NMSC), predominantly basal cell carcinoma (BCC) and squamous cell carcinoma (SCC), represents the most common malignancy among fair-skinned populations. While BCC is rarely fatal, SCC contributes significantly to NMSC-related mortality. This study aimed to investigate long-term trends in NMSC incidence and SCC mortality across 28 European countries from 1992 to 2021, focusing on regional, sex-specific, and age-related variations.

**Methods/patients:**

A longitudinal ecological analysis was conducted using the Global Burden of Disease (GBD) database. Age-standardized incidence rates (ASIRs) for NMSC and age-standardized mortality rates (ASMRs) for SCC were calculated based on the 2013 European Standard Population. Temporal trends were evaluated using joinpoint regression, and age–period–cohort (APC) models were employed to disentangle independent effects on SCC mortality.

**Results:**

Europe registered over 27 million NMSC cases between 1992 and 2021. Overall ASIRs slightly declined, although increasing incidence was observed in individuals under 45 in Central and Northern Europe. SCC accounted for more than 570,000 deaths, with overall ASMRs decreasing—particularly among women and younger men. However, mortality rose in men aged over 75, notably in Northern and Western Europe. APC analysis indicated elevated SCC mortality in cohorts born before 1940, with a notable reversal in Northern European males born after 1960, who exhibited increasing mortality. Period effects further confirmed a recent rise in SCC mortality among these populations.

**Conclusions:**

Although NMSC incidence appears to be stabilizing or declining in much of Europe, increasing trends in younger individuals and rising SCC mortality in older men—especially in Northern Europe—highlight the need for age- and region-specific prevention and screening strategies. Improved cancer registry harmonization remains essential for guiding effective public health interventions.

**Graphical abstract:**

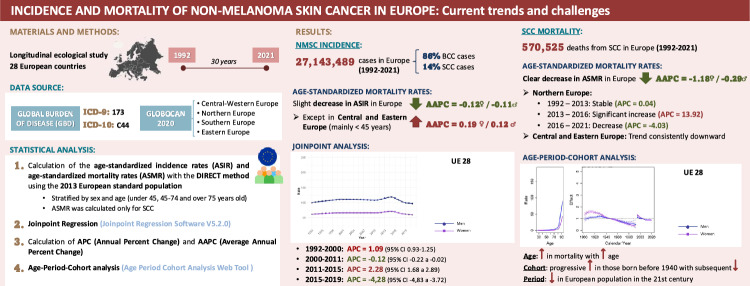

**Supplementary Information:**

The online version contains supplementary material available at 10.1007/s12094-025-03985-z.

## Introduction

Nonmelanoma skin cancer (NMSC) is the most common type of cancer among fair-skinned populations worldwide [1]. This group of neoplasms mainly includes basal cell carcinoma (BCC) and squamous cell carcinoma (SCC), although it also comprises less common tumors, such as Merkel cell carcinoma [2]. Despite its high prevalence, mortality associated with NMSC has historically been low, particularly in the case of BCC [3]. However, SCC presents a different pattern, with increasing mortality rates in certain population subgroups, such as immunocompromised individuals and older adults [2].

In Europe, NMSC incidence rates have shown a steady increase in the recent decades [4], with projections indicating a potential increase of approximately 40% in the coming decades, driven by factors such as UV exposure and demographic changes, particularly the aging population [5]. This increase is particularly notable in Northern European countries, where NMSC incidence far exceeds that of southern regions. According to available data, incidence varies from more than 100 cases per 100,000 people in some regions to fewer than 50 cases per 100,000 in others, highlighting significant geographic disparities within the continent [6].

Although the incidence of NMSC continues to rise, mortality rates have remained relatively stable, reflecting improvements in early diagnosis and the development of nonoperative treatment modalities [7]. However, slight increases have been observed in specific subgroups, notably among women born after the 1970s in certain countries [8]. These differences reflect both genetic and environmental factors, including the effectiveness of early detection programs and treatment strategies [9, 10].

At the continental level, the quality of epidemiological data and registry systems varies significantly, hindering direct comparisons between countries. Although some nations have robust NMSC data collection systems, others face limitations in standardization and the availability of information disaggregated by tumor subtypes. These disparities represent a major challenge in designing effective prevention policies [4, 11–15].

This study aims to comprehensively analyze NMSC incidence and mortality trends in 28 European countries from 1992 to 2021. For mortality analysis, the study will focus only on SCC as it accounts for the majority of NMSC-related deaths, as highlighted in previous studies [8, 16, 17]. By exploring variations by gender, age, and geographic region, the study aims to provide a more complete perspective on the disease's impact throughout Europe, thereby contributing valuable insights for the development of effective public health strategies.

## Materials and methods

A longitudinal ecological study was performed to investigate patterns in the incidence and mortality of nonmelanoma skin cancer (NMSC) across 28 European countries over the period 1992 to 2021. Information on NMSC incidence and mortality was obtained from the Global Burden of Disease (GBD) database (https://ghdx.healthdata.org/). BCC and SCC cases were specifically identified using ICD-9 code 173 and ICD-10 code C44, covering all individuals (both sexes) in the selected countries. The countries were categorized into four geographic regions—Central and Eastern Europe, Northern Europe, Southern Europe, and Western Europe—according to the GLOBOCAN 2020 classification [18]. To ensure reliable reporting of health estimates, the study adhered to the GATHER (Guidelines for Accurate and Transparent Health Estimate Reporting) [19] standards and followed the STROBE (Strengthening the Reporting of Observational Studies in Epidemiology) [20] guidelines for observational research.

### Statistical analysis

Incidence and mortality data were divided into three age groups: under 45 years, 45 to 74 years, and over 75 years. This stratification was based on previous research that supports this classification due to differences in disease latency and biological factors [21].

Age-standardized incidence rates (ASIRs) and age-standardized mortality rates (ASMRs) were determined using the direct standardization method, referencing the 2013 European Standard Population [22]. ASMRs for BCC were not calculated due to its residual mortality [2, 8, 23]. ASIRs and ASMRs were reported separately by sex and age group and expressed as rates per 100,000 individuals. The data processing and analysis were conducted with R statistical software (version 4.3.2; https://www.r-project.org/).

Trends in ASIRs and ASMRs over time were evaluated using Joinpoint regression software (version 5.2.0.0; https://surveillance.cancer.gov/joinpoint). This method calculated both the annual percentage change (APC) and the average annual percentage change (AAPC) for each segment of the study period (1992–2021). The rate changes were deemed statistically significant if the APC or AAPC was greater or less than zero (p < 0.05). Stable trends were identified when no significant change was observed. A maximum of 7 joinpoints was permitted, and pairwise comparisons were performed to determine if trends differed between men and women.

The independent effects of age, period, and cohort (age–period–cohort analysis, A–P–C) on mortality rates were assessed using the cohort analysis tool developed by the National Cancer Institute (https://analysistools.cancer.gov/apc/) [24]. Wald tests were applied to evaluate the statistical significance of the findings. Statistical significance was considered when the *p* value was less than 0.05.

## Results

### NMSC incidence

Durante the study period from 1992 to 2021, Europe recorded an estimated total of 27,143,489 cases of NMSC, with 23,356,291 BCC cases (86%) and 3,787,198 SCC (14%) cases.

A slight decrease in ASIRs was observed at the global level in Europe for women (AAPC = −0.12; 95% CI −0.22 to -0.02) during the study period (Table 1). However, not all regions followed this trend, with the analysis showing a slight increase in incidence in Central and Eastern Europe for both men (AAPC = 0.12; 95% CI 0.05 to 0.20) and women (AAPC = 0.19; 95% CI 0.03 to 0.35). When stratifying the data by age, it is noteworthy that this increase was primarily driven by the younger population (< 45 years) (AAPC = 0.20; 95% CI 0.14 to 0.26 for men; and = 0.26; 95% CI 0.13 to 0.39 for women) (Supplementary Table 1 ). Detailed information on the ASIRs of NMSC from the 28 European countries included in the study in ≥ 45 years old can be found in Supplementary Tables 2 and 3.

**Table 1:**
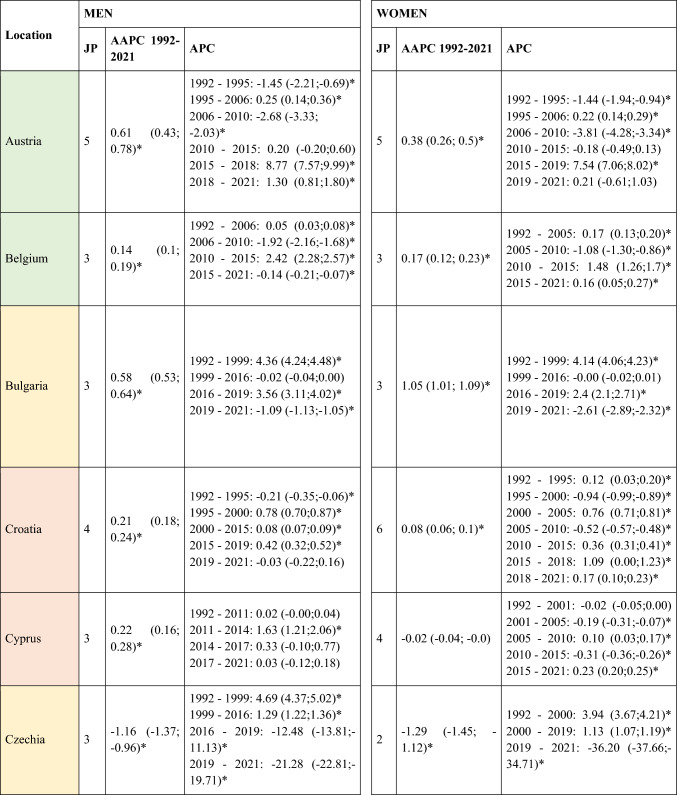

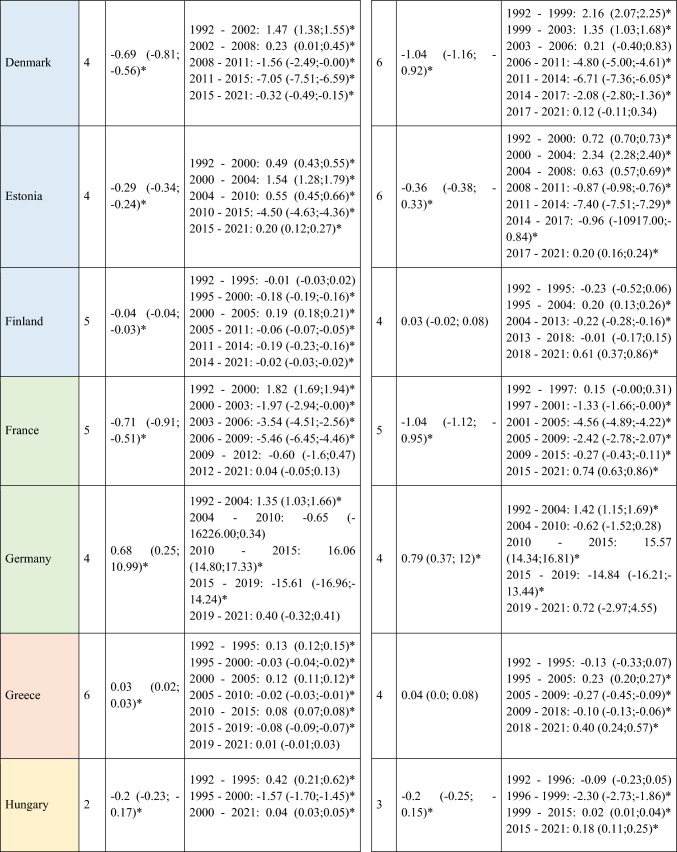

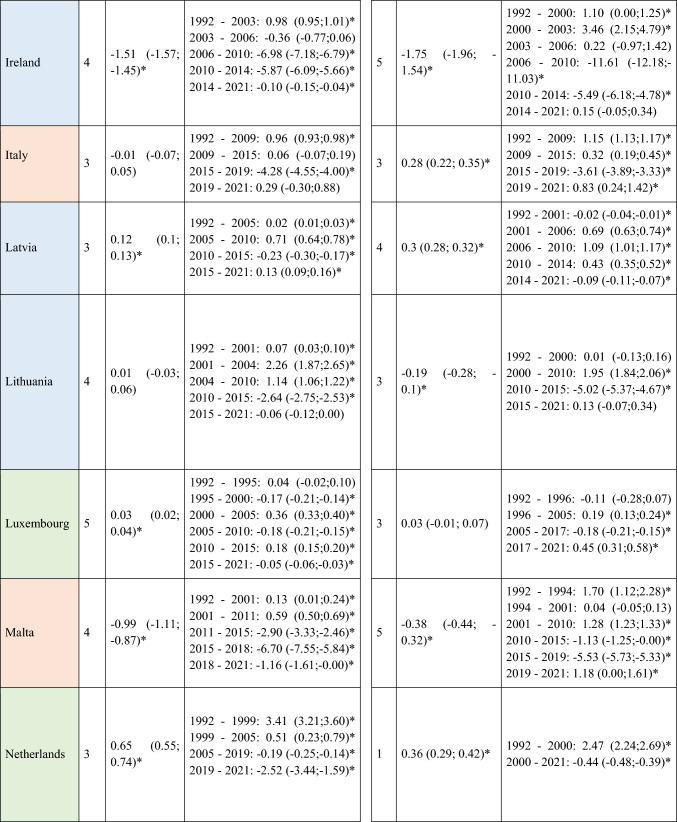

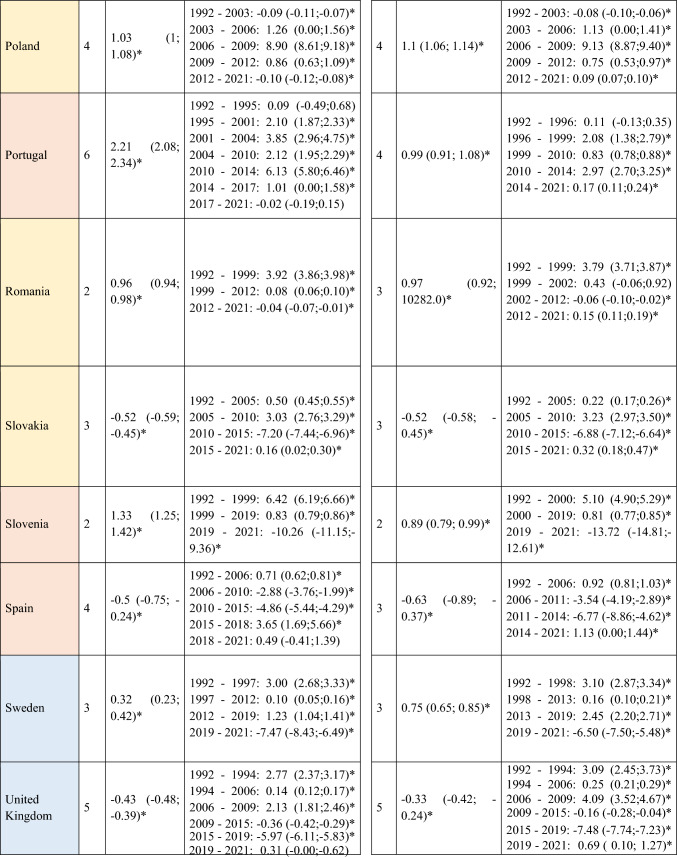

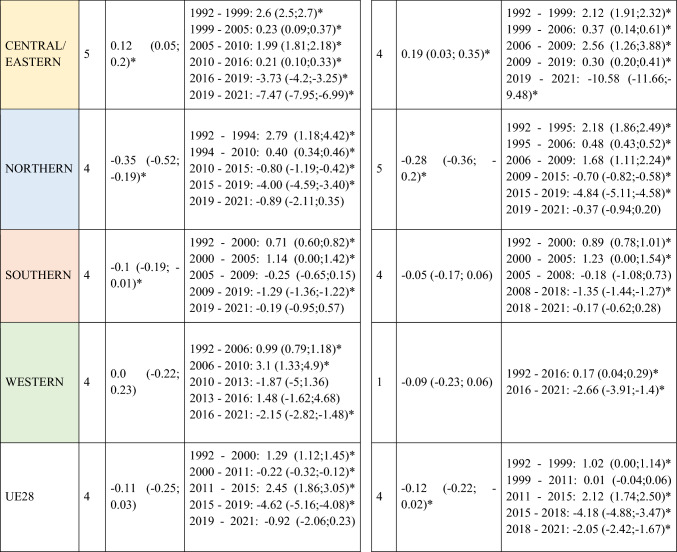
Joinpoint Analysis Results for NMSC Incidence in European countries (1992–2021) by sex

*AAPC* Anual Average percentage change. *JP* Joinpoint. *APC* Annual Percentage Change and 95% confidence interval. *p < 0.05

Western countries: green, Southern countries: red, Northern countries: blue, Central and Eastern countries: yellow

**Fig. 1:**
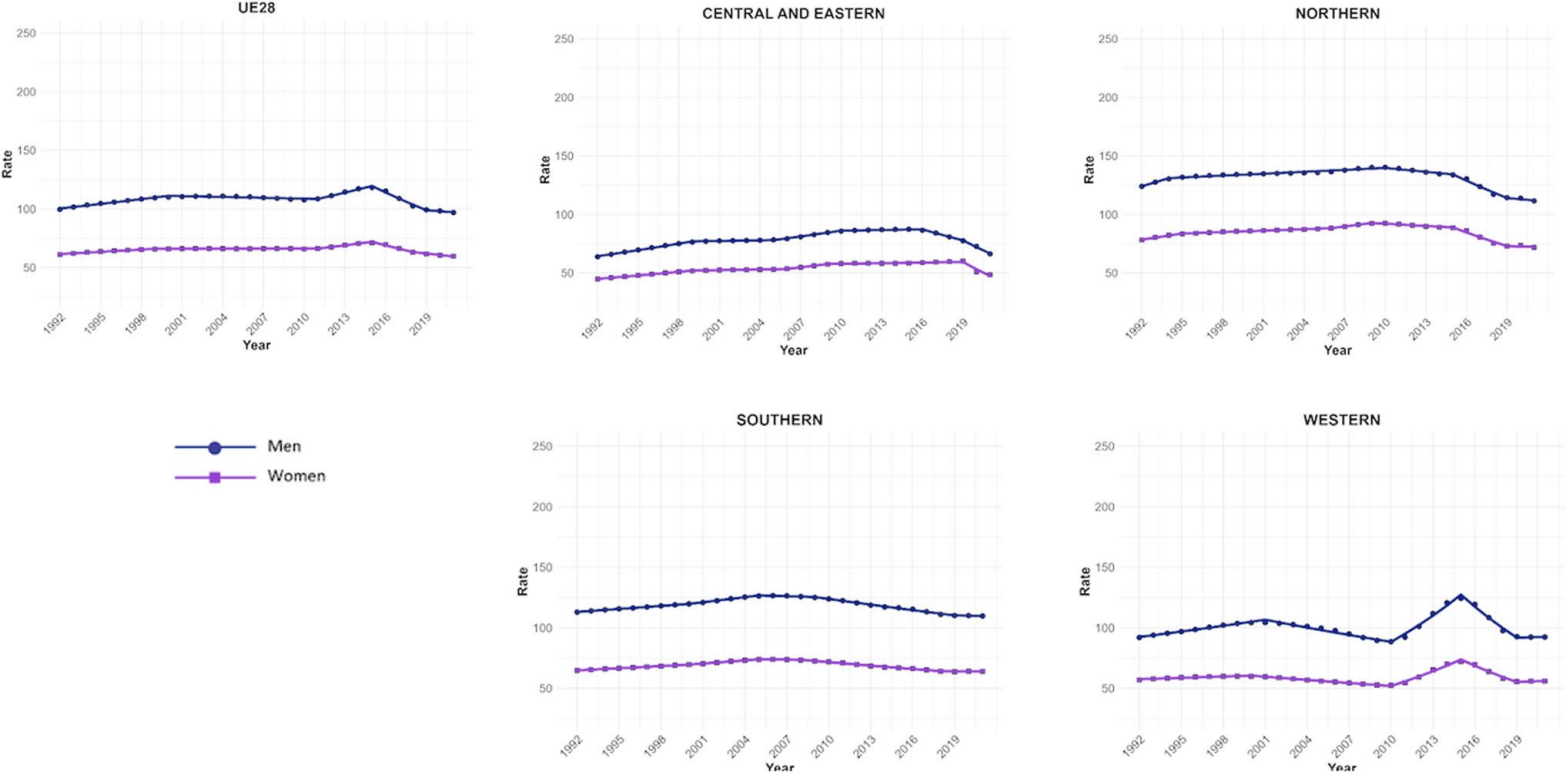
Joinpoint Regression Analysis of NMSC Incidence for Both Sexes in Four European Regions (1992–2021)

Four trend changes in NMSC incidence were observed in Europe (Fig. 1). The first phase showed an increase from 1992 to 2000 (APC = 1.09; 95% CI 0.93 to 1.25), followed by a slight decline until 2011 (APC = −0.12; 95% CI −0.22 to -0.02). This was succeeded by a significant rise until 2015 (APC = 2.28; 95% CI 1.68 to 2.89), after which a marked decline was observed (APC = −4.28; 95% CI −4.83 to −3.72). This general decline appears to have stabilized since 2019. However, when stratifying the data by regions, we observe that in young patients (< 45 years) from Northern Europe, ASIRs do not follow this trend. Instead, they show an increase since 2019, both in men (APC = 0.92; 95% CI 0.65 to 1.2) and in women (APC = 0.49; 95% CI 0.18 to 0.79) (Supplementary Table 1and Supplementary Fig. 1. This increase in NMSC incidence in Northern Europe since 2019 was not observed in older patients. In fact, a significant decline in NMSC incidence was found in men over 75 years (APC = −2.3; 95% CI −3.04 to −1.55), as well as in women (APC = −1.79; 95% CI −2.99 to −0.58) (Supplementary Table 3).

### SCC mortality

A total of 570,525 deaths from SCC occurred in Europe between 1992 and 2021. Stratifying by sex, at the European level, the ASMRs for SCC showed a significant decrease in women, from 1.31 cases per 100,000 inhabitants during the period 1992–1996 to 1 case per 100,000 inhabitants in 2017–2021 (AAPC = −1.18; 95% CI −1.41 to −0.95) (Table 2). Among men, this decrease was observed in those under 45 years old (AAPC = −1.76; 95% CI −2.31 to −1.21), and in those aged 45 to 74 years (Supplementary Tables 4 and 5). When analyzed by region, the data revealed that this decline in ASMRs was not uniform. Men in Western Europe exhibited an overall increase in rates (AAPC = 0.65; 95% CI 0.17 to 11.32) (Table 2), which was also observed in women (AAPC = 0.74; 95% CI 0.16 to 13.24) and in men from Northern Europe (AAPC = 1.15; 95% CI 0.15 to 2.17) aged > 74 years (Supplementary Table 6).

**Table 2:**
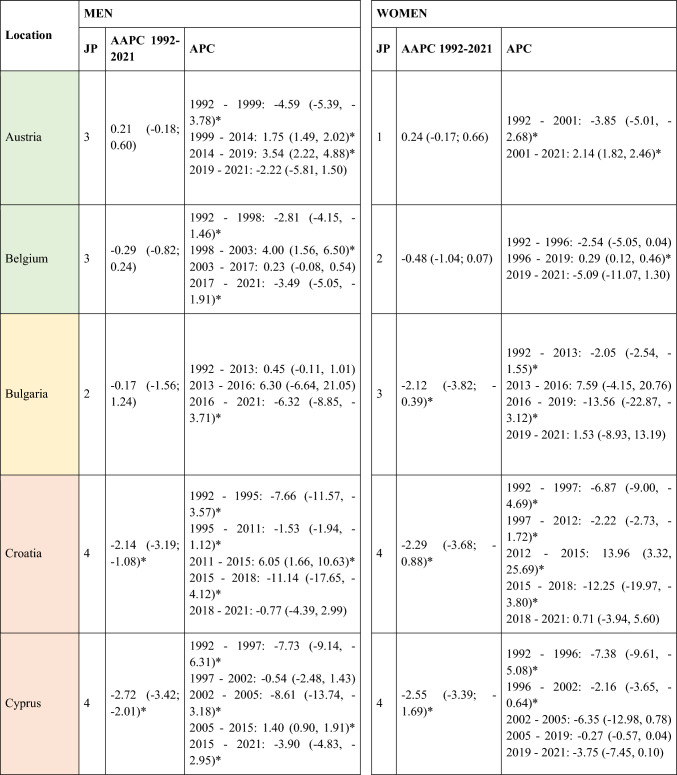

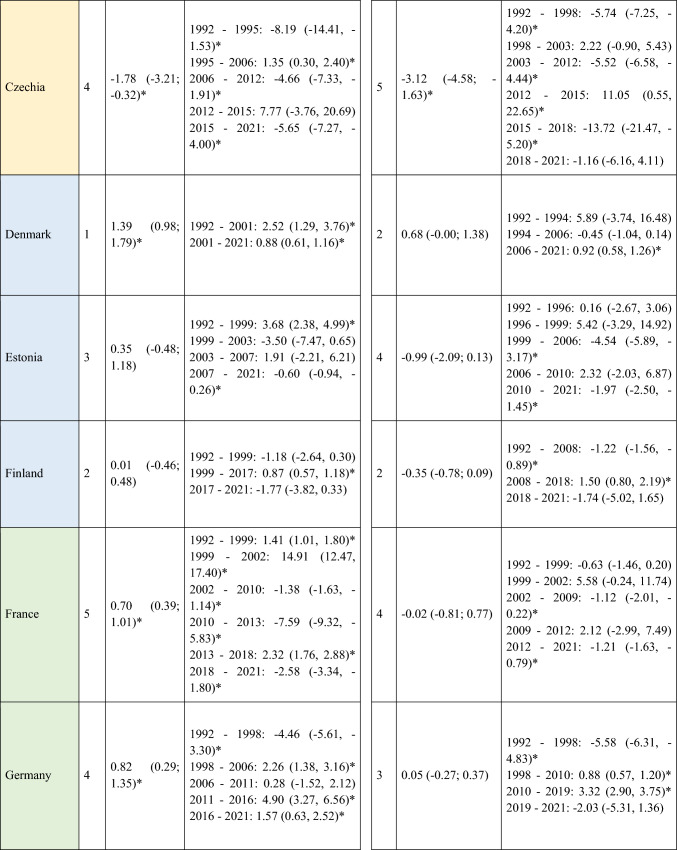

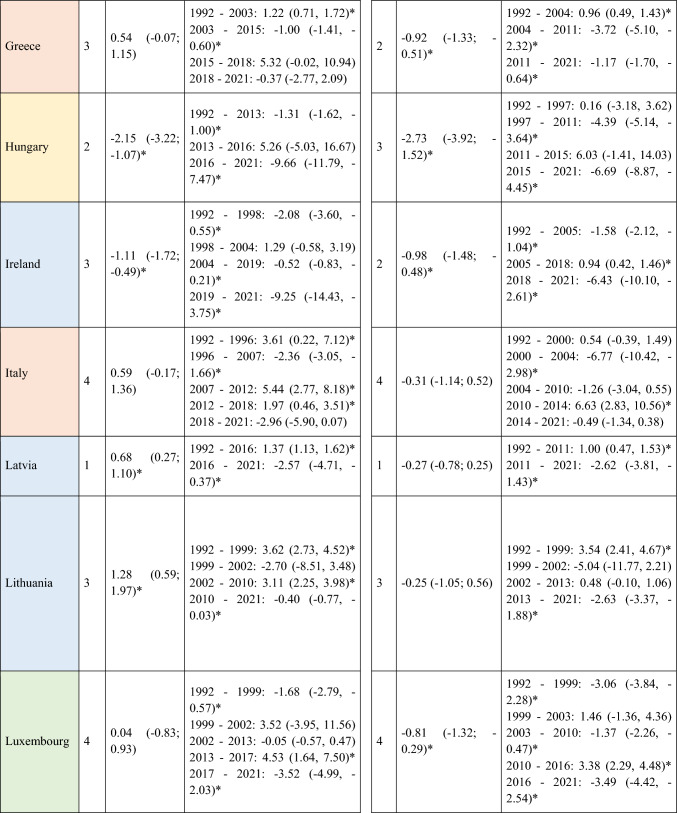

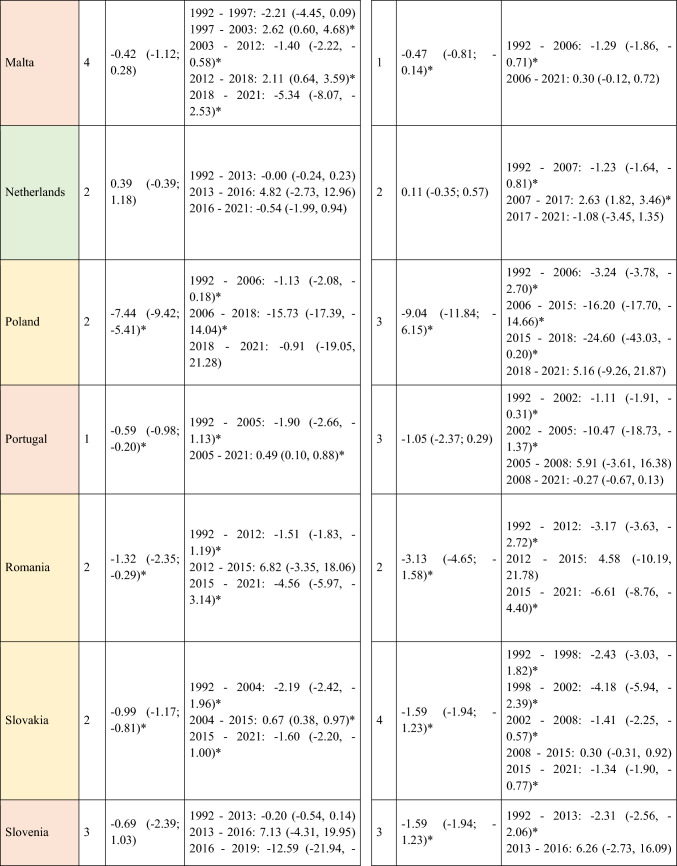

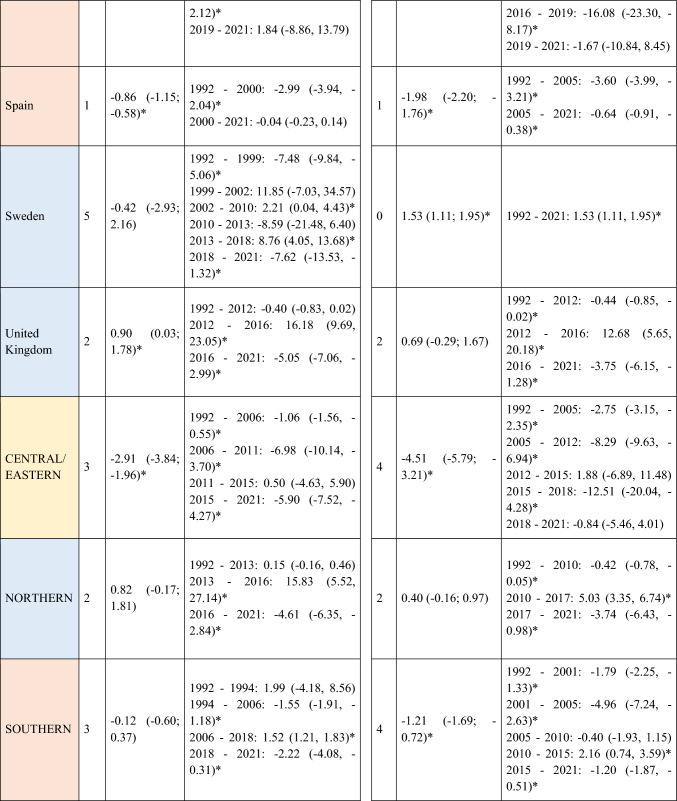

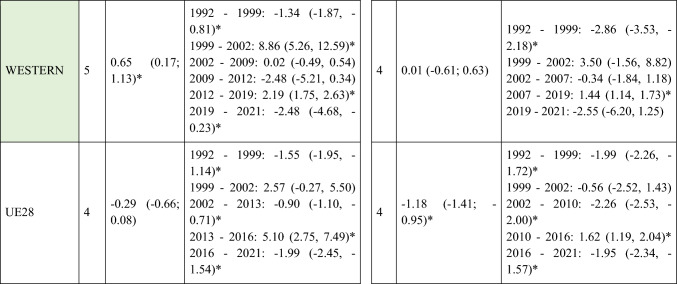
Joinpoint Analysis Results for SCC Mortality in European countries (1992–2021) by sex

*AAPC* Annual Average percentage change. *JP* Joinpoint. *APC* Annual Percentage Change and 95% confidence interval. *p < 0.05

Western countries: green, Southern countries: red, Northern countries: blue, Central and Eastern countries: yellow

**Fig. 2:**
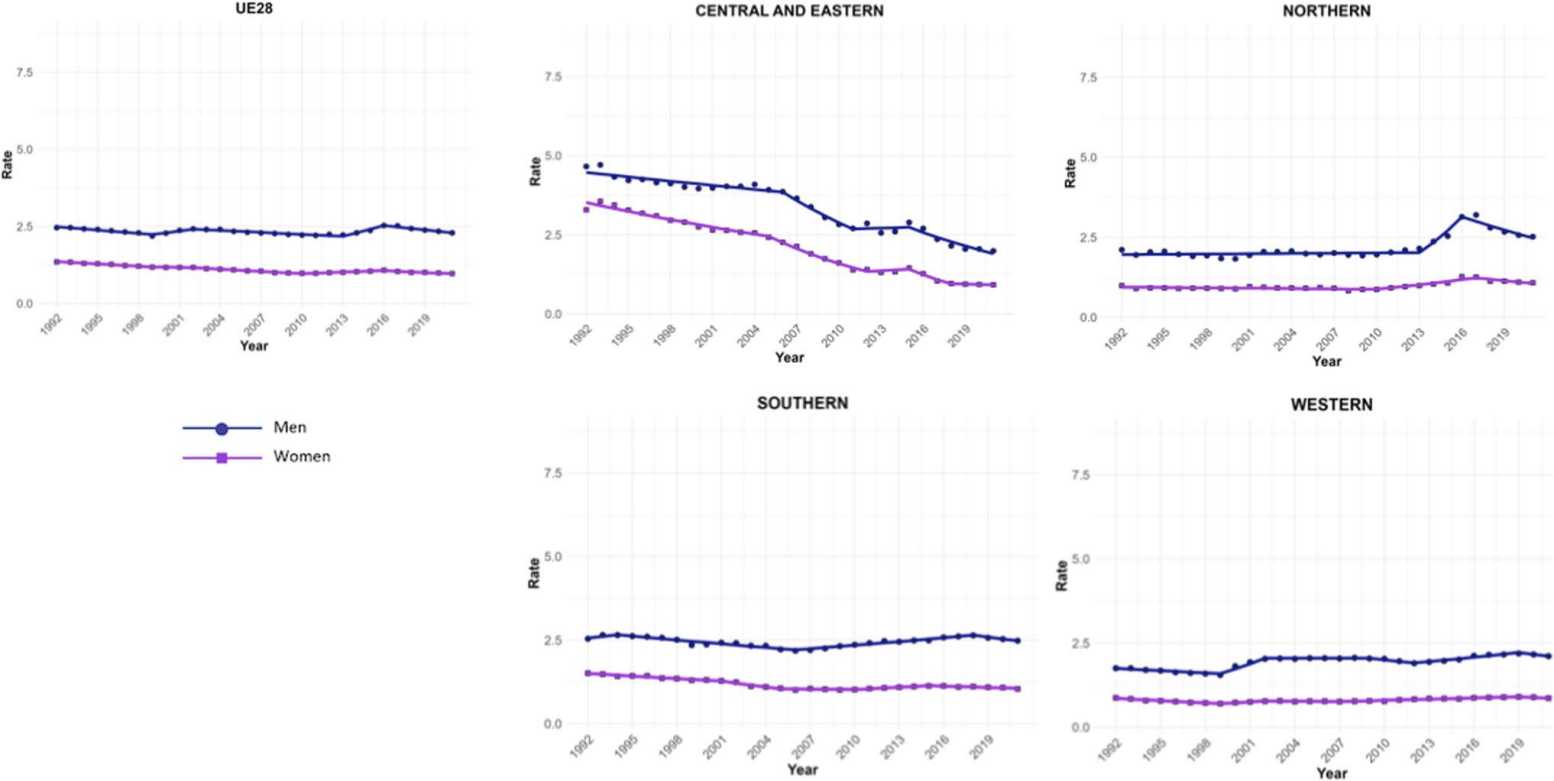
Joinpoint Regression Analysis of SCC Mortality for Both Sexes in Four European Regions (1992–2021)

**Fig. 3:**
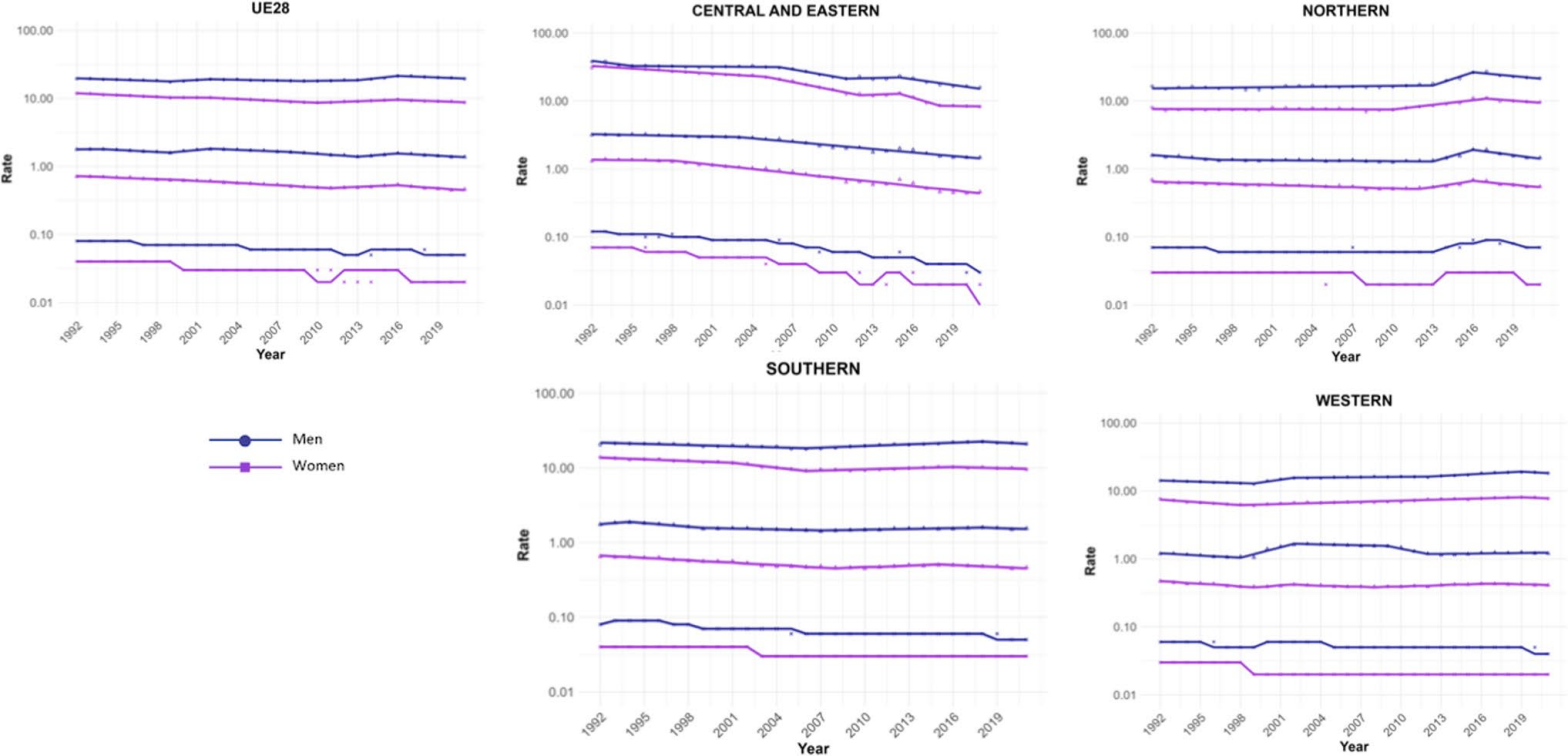
Joinpoint Regression Analysis of SCC Mortality for Both Sexes in Age Groups in Four European Regions (1992–2021)

For Northern Europe, the analysis revealed a highly significant trend change in mortality, with three distinct periods (Fig. 2). Initially, there was a stabilization phase in mortality from 1992 to 2013 (APC = 0.04; 95% CI −0.2 to 0.46), followed by a highly significant increase from 2013 to 2016 (APC = 13.92; 95% CI 5.44 to 23.09), and a subsequent decline from 2016 to 2021 (APC = −4.03; 95% CI −5.51 to −2.52). When stratifying the data by sex, it was observed that the marked increase in mortality between 2013 and 2016 was predominantly driven by males (APC = 15.83; 95% CI 5.52 to 27.14), whereas females also showed an increase, though less pronounced (APC = 5.03; 95% CI 3.35 to 6.74) and over a broader period (2010–2017). Moreover, when stratifying the data by age (Fig. 3), the three age groups studied exhibited the same trends, but the increase was particularly pronounced in males aged > 74 years (APC = 16.02; 95% CI 5.57 to 27.51).

Conversely, in Central and Eastern Europe, the trend was consistently downward throughout the entire period (Fig. 3), with a trend change observed from 2004, showing a more significant decline thereafter (APC = −4.96; 95% CI −5.71 to v4.21). When segregating the data by sex, the downward trend was observed in both groups but became particularly pronounced in women after 2015 (APC = −12.6; 95% CI −20.33 to −4.12).

**Fig. 4:**
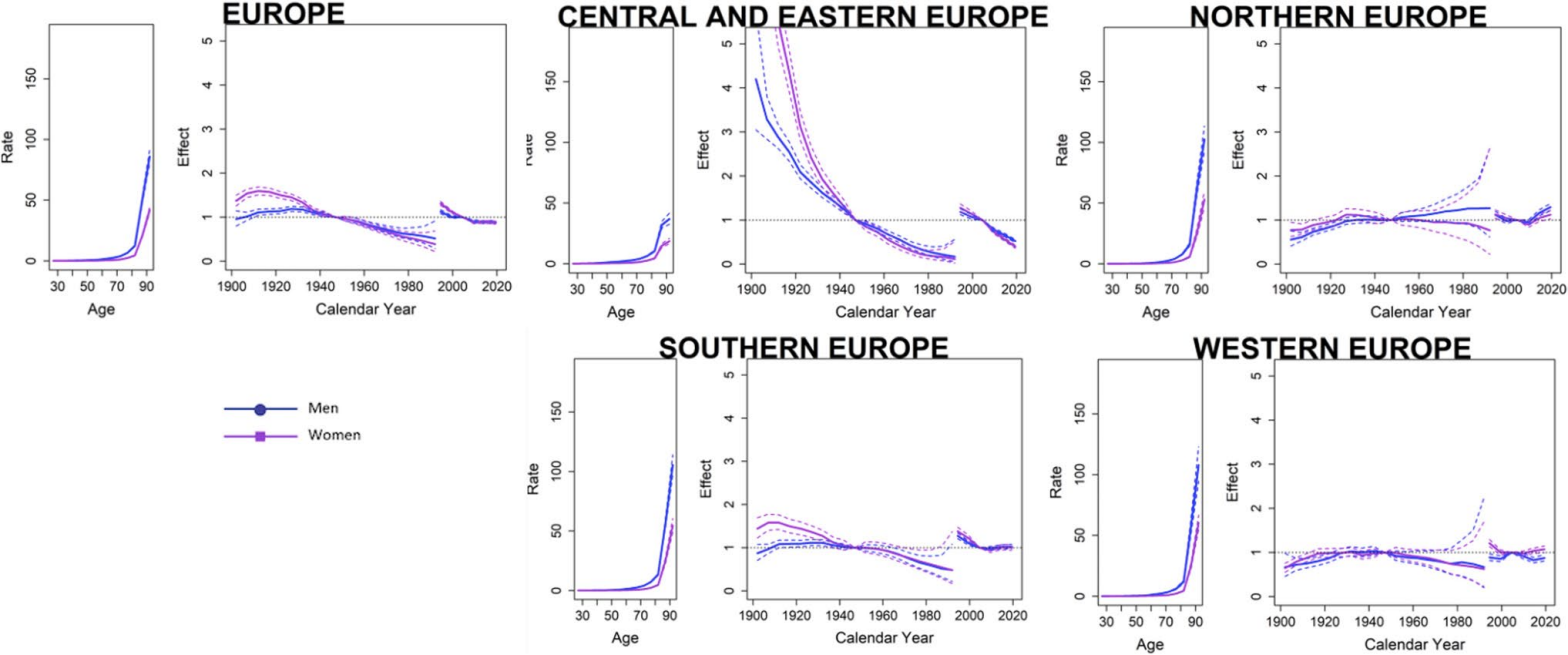
Age–period–cohort effect analysis on NMSC Mortality for Both Sexes in Four European Regions

Regarding the A–P–C analysis (Fig. 4), a progressive increase in mortality rates with age was observed. Concerning the birth cohort effect, individuals born at the beginning of the century (particularly women) exhibited higher ASMRs, which progressively declined for those born from 1940 onward. When breaking down the data by region, mortality was notably high among both women and men from Central and Eastern Europe born at the beginning of the century. A similar, although less pronounced, increase in mortality was observed among women from Southern Europe born during the same period. A reduction in risk was evident for cohorts born after 1940 in Central and Eastern Europe, Southern Europe, and Western Europe. However, in Northern Europe, the trend was reversed, with the male cohort born after 1960 showing higher rates compared to those born earlier in the century.

Finally, regarding the period effect, a general downward trend in ASMRs has been observed among European men and women during the twenty-first century. Analyzing by regions, the exception to this trend is Northern Europe, where rates for both sexes have tended to rise, particularly among males, with these increases being especially pronounced in countries such as the United Kingdom (Supplementary Fig. 2). Detailed results for the 28 countries included in the mortality analysis can be found in the supplementary material (Supplementary Figs. 1, 2, 3, 4, and Supplementary Tables 1, 2, 3, 4, 5, 6).

## Discussion

The present study offers a comprehensive analysis of the incidence and mortality trends of NMSC across 28 European countries over three decades. The findings highlight significant geographic and demographic disparities, underscoring unique regional variations.

According to our results, the ASIRs of NMSC in Europe showed a slight overall decline. When stratifying the data by regions, a slight increase in NMSC incidence was observed in Central and Eastern Europe for both sexes. The rise in incidence was particularly notable among patients younger than 45 years. Similar trends had already been observed in other parts of Europe. Birch–Johansen et al. [25] observed a significant increase in the incidence of BCC in young women (< 40 years) in Denmark between 1978 and 2007. Also, an increase in BCC has been reported in Iceland among young women between 1982 and 2007 [26]. Similarly, Cayuela et al. [27] found a generalized decline in ASIRs in Spain; however, young cohorts revealed that young men (20–24 years) did not show a significant decrease in incidence. These increases may be attributed to changes in sun exposure behaviors or practices such as tanning beds, which contribute to the rise in skin cancer incidence and are more frequent in younger groups [6, 28]. Outdoor occupations among younger individuals—such as those in sports or tourism—may also contribute to regional differences in incidence due to higher cumulative exposure to ultraviolet radiation at earlier ages. According to our results, since 2019 the overall increase in the incidence of NMSC in Europe seems to have stabilized. However, among young people in Northern Europe, this upward trend has continued among men and also among women, indicating the importance of a tailored approach to public health policies aimed at this age group in these European regions. Several behavioral and systemic factors may explain the observed differences in NMSC trends across Europe. The increased incidence among younger individuals, particularly in Northern and Central Europe, may reflect changes in recreational UV exposure habits, including higher use of tanning beds and increased travel to sunny destinations. These behaviors have been previously linked to higher NMSC risk, especially among younger women. Moreover, disparities in access to dermatological care, availability of organized skin cancer screening programs, and levels of diagnostic awareness among both the general population and healthcare professionals could contribute to regional variations in both incidence and mortality. Countries with robust screening initiatives may report higher incidence due to earlier detection, whereas regions with limited access to specialists or public health campaigns may experience underdiagnosis or delayed treatment, potentially impacting mortality trends. Increased public awareness, policy regulations (such as restrictions on indoor tanning), and expanded skin cancer screening initiatives may partially account for the observed declines in ASIRs in some European regions. Public education campaigns promoting UV protection, skin self-examination, and early consultation with healthcare professionals have contributed to greater vigilance and early detection. To optimize these strategies for high-risk populations—such as younger individuals with high UV exposure or older adults with occupational risk—tailored interventions are needed.

Previous studies show that, of the total deaths from NMSC, the majority should be considered deaths from SCC, given that BCC mortality is minimal [8, 16, 17]. There are few studies in the literature on NMSC-related mortality, as it is not consistently included in registries [4, 11]–[14]. According to our results, a general decline in ASMRs was observed in both men and women. Previous studies have reported a slight decline in NMSC-related ASMRs in European countries such as the Netherlands [29] and Finland [30], as well as in non-European countries like the United States [31]. On the contrary, in a more recent study Aggarwal et al. [15] showed a slight increase in NMSC mortality in the United States.

Improvements in early detection and treatment likely play a significant role in the observed decline in SCC mortality among women and younger men. Greater awareness of skin cancer symptoms, increased health-seeking behavior—particularly among women—and widespread access to dermatological care may lead to earlier diagnosis and more effective management.

In our study, the exception to the general decline of NMSC ASMRs in Europe was observed in males over 74 years of age. In contrast, Leiter et al. [13] reported a decline in ASMRs among men over 74 in Germany. However, this previous study only included ASMRs data up to 2012. Based on our findings, a trend reversal in ASMRs among men over 75 years of age in Germany began in 2010, followed by a particularly marked increase and a sustained rise in rates throughout the study period (1992–2021).

According to our results, data regarding males from Northern European countries are particularly concerning, as they reveal an increasing trend in mortality. Males born after 1960 exhibit higher ASMRs compared to those born earlier in the century in our study. Moreover, a period effect indicates a continued rise in ASMRs among Northern European males in recent years. This concerning rise in SCC mortality among older men, particularly in Northern and Western Europe, is likely multifactorial. In addition to cumulative ultraviolet radiation exposure and age-related immunosenescence, limited engagement in preventive health behaviors—such as self-examination and regular skin checks—may delay diagnosis in this population. Furthermore, healthcare access barriers, especially in rural or underserved areas, could contribute to diagnostic and treatment delays. Together, these factors may result in more advanced-stage presentations and poorer outcomes in older men, underscoring the need for targeted interventions in this demographic. In 2017, Brunssen et al. [10] conducted a systematic review to analyze the role of skin cancer secondary prevention. The results showed that screening is associated with an increase in the incidence of NMSC; however, evidence regarding its impact on mortality from this disease is limited, highlighting the need for further studies on the subject. In this context, the role of secondary prevention in specific high-risk groups, such as those highlighted in this study, could be of particular interest in improving mortality data [32, 33].

The main limitation of this study is that the data from the GBD are based on estimates and mathematical models derived from various sources, meaning not all cases of skin cancer are likely to have been recorded. Moreover, heterogeneity in data quality, reporting standards, diagnostic practices, and case coding across the 28 European countries included in this study may affect the accuracy of country-specific estimates. The completeness and frequency of updates to national cancer registries also vary, further limiting comparability. As noted in the previous studies, underreporting and inconsistent classification of NMSC cases remain significant challenges [4, 11–15].

In conclusion, although the overall incidence of BCC has shown a downward trend in Europe, our data reveal an increase among young patients in Central, Eastern, and Northern Europe. SCC incidence, in turn, has exhibited a modest rise among European males. On the other hand, SCC mortality also shows a decreasing trend, although heterogeneously, with specific increases in certain groups, such as men from Northern European countries, especially those born after the 1960s. These findings underscore the need for tailored public health strategies. In clinical practice, enhanced vigilance is warranted for younger patients in Northern and Central Europe and for elderly men, particularly in regions with rising SCC mortality. Public health authorities should prioritize harmonized registry systems to improve data comparability, while also developing region-specific prevention campaigns, including UV protection education and potential screening initiatives in high-risk populations. Integrating these epidemiological insights into clinical and policy frameworks may help reduce the future burden of NMSC across Europe.

## Supplementary Information

Below is the link to the electronic supplementary material.

Supplementary file1 Supplementary Figure 1. Joinpoint Regression Analysis of NMSC Incidence by Age for Both Sexes in Four European Regions (JPG 580 KB)

Supplementary file2 Supplementary Figure 2. Age-Period-Cohort Effect Analysis on SCC Mortality for Both Sexes in the 28 European Countries Studied (JPG 627 KB)

Supplementary file3 Supplementary Figure 3. Joinpoint Regression Analysis of SCC Mortality for Both Sexes in the 28 European Countries Studied (JPG 388 KB)

Supplementary file4 Supplementary Figure 4. Joinpoint Regression Analysis of SCC Mortality by Age Groups for Both Sexes in the 28 European Countries Studied (JPG 578 KB)

Supplementary file5 (DOCX 23 KB)

Supplementary file6 (DOCX 22 KB)

Supplementary file7 (DOCX 23 KB)

Supplementary file8 (DOCX 22 KB)

Supplementary file9 (DOCX 21 KB)

Supplementary file10 (DOCX 23 KB)

## Data Availability

The data for this study are publicly available through the Global Burden of Disease database (https://ghdx.healthdata.org/).
