# Supplementary figures and images for: Incidence and mortality of nonmelanoma skin cancer in Europe: current trends and challenges
Source: Clin Transl Oncol. 2025 Jul 11;28(1):302–19. doi: 10.1007/s12094-025-03985-z (PMC12790528; doi:10.1007/s12094-025-03985-z)

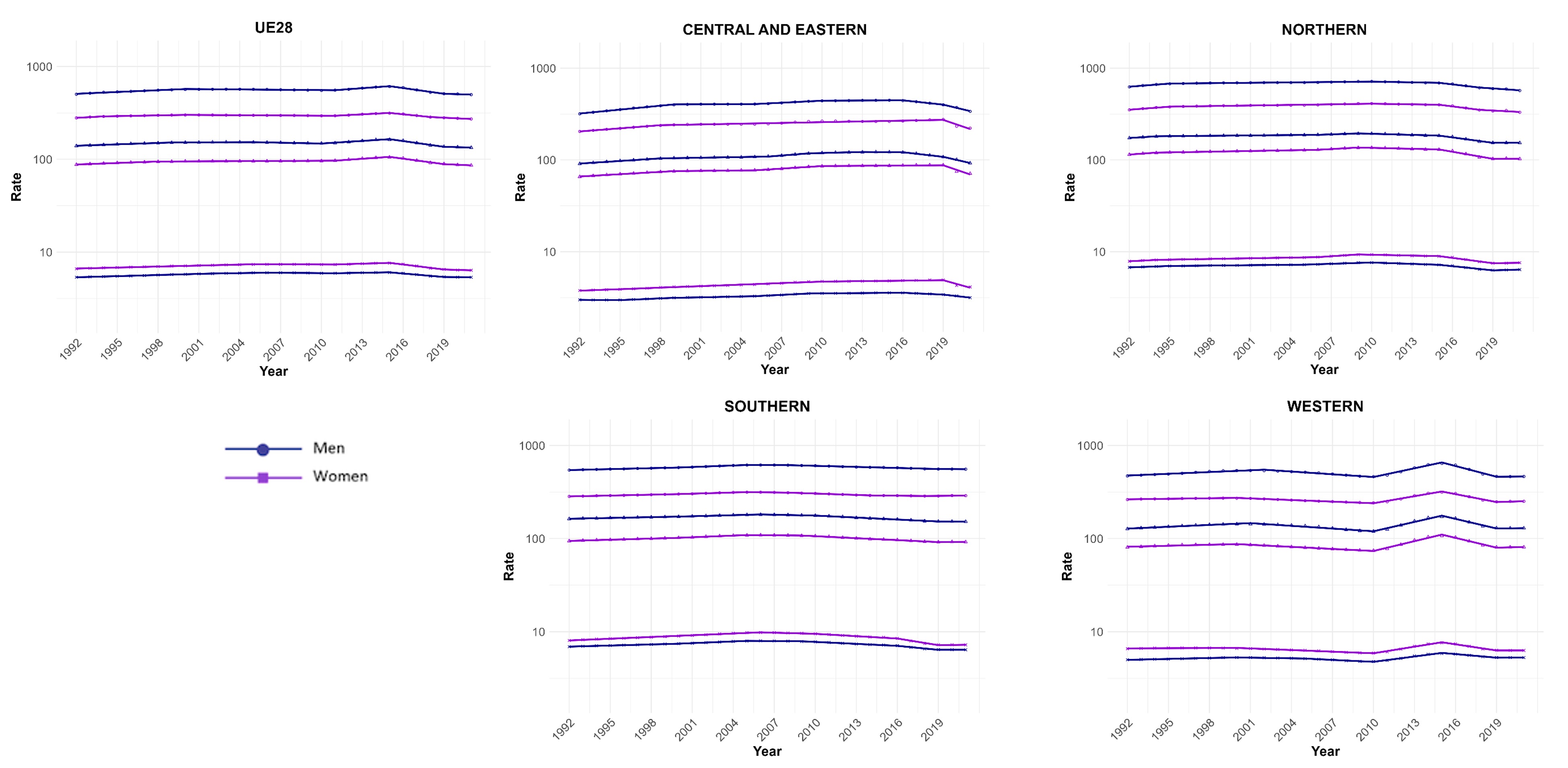

Supplement: Supplementary file 1 — Supplementary file1 Supplementary Figure 1. Joinpoint Regression Analysis of NMSC Incidence by Age for Both Sexes in Four European Regions (JPG 580 KB) [file 12094_2025_3985_MOESM1_ESM.jpg]

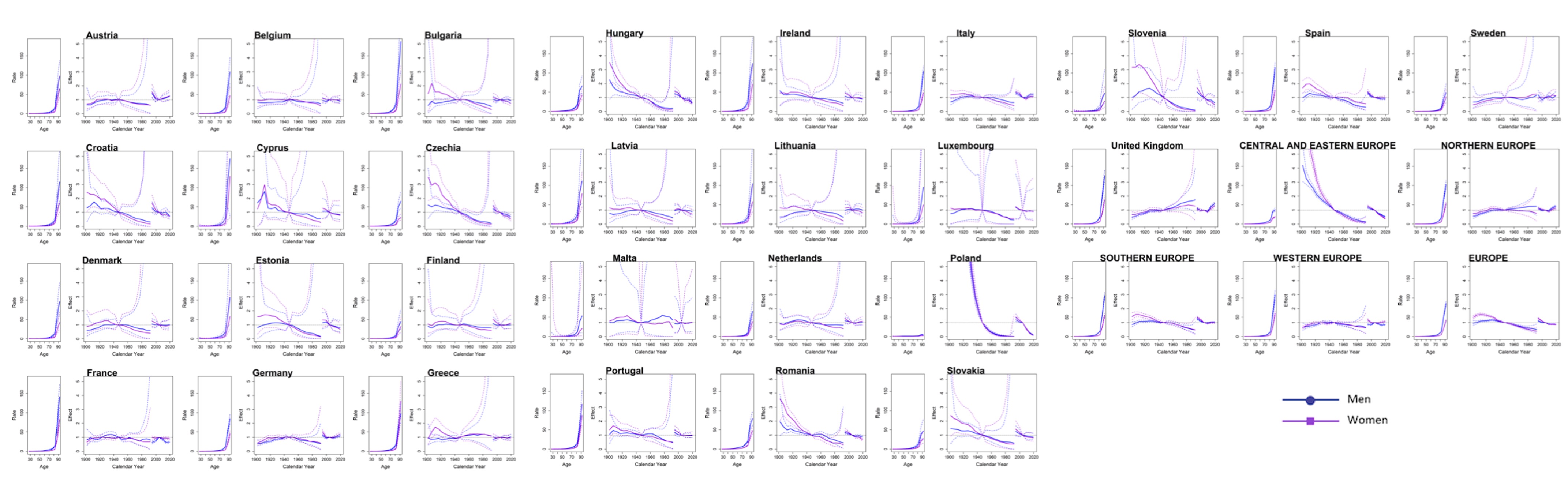

Supplement: Supplementary file 2 — Supplementary file2 Supplementary Figure 2. Age-Period-Cohort Effect Analysis on SCC Mortality for Both Sexes in the 28 European Countries Studied (JPG 627 KB) [file 12094_2025_3985_MOESM2_ESM.jpg]

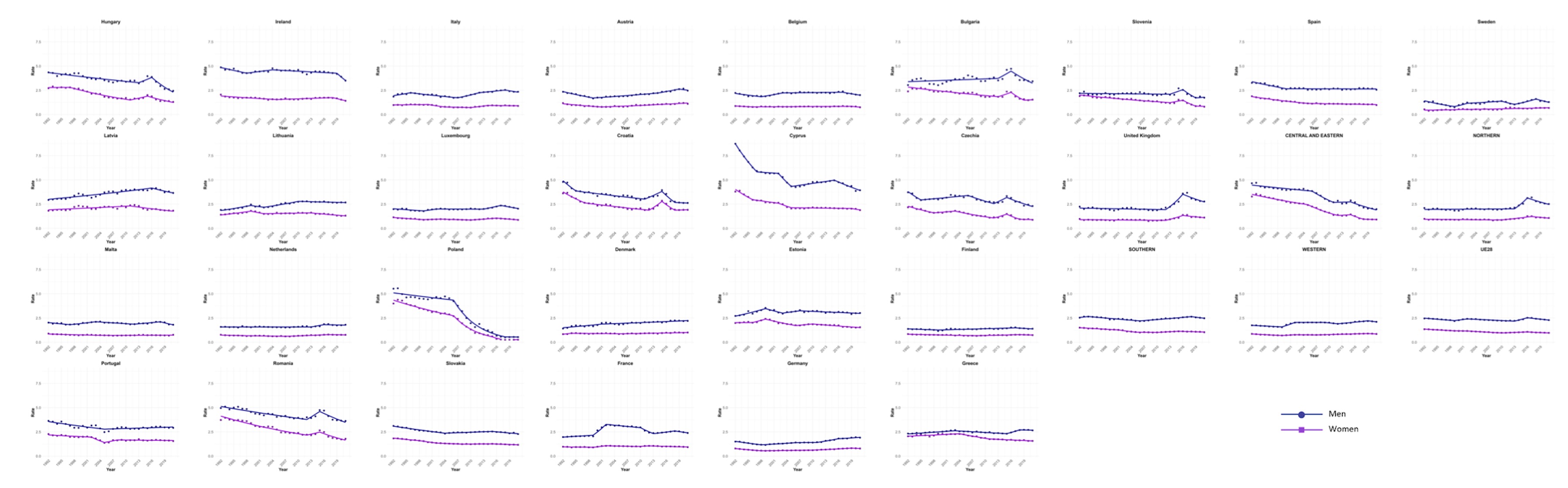

Supplement: Supplementary file 3 — Supplementary file3 Supplementary Figure 3. Joinpoint Regression Analysis of SCC Mortality for Both Sexes in the 28 European Countries Studied (JPG 388 KB) [file 12094_2025_3985_MOESM3_ESM.jpg]

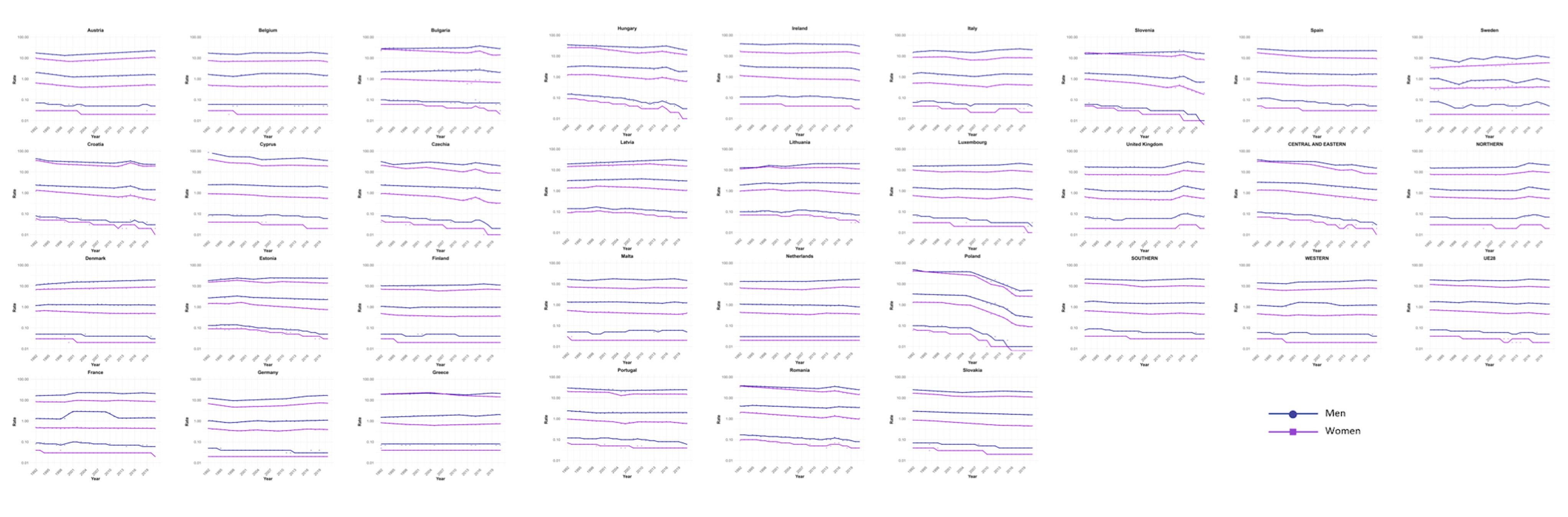

Supplement: Supplementary file 4 — Supplementary file4 Supplementary Figure 4. Joinpoint Regression Analysis of SCC Mortality by Age Groups for Both Sexes in the 28 European Countries Studied (JPG 578 KB) [file 12094_2025_3985_MOESM4_ESM.jpg]
