# Supplementary material for: Incidence and mortality of nonmelanoma skin cancer in Europe: current trends and challenges
Source: Clin Transl Oncol. 2025 Jul 11;28(1):302–19. doi: 10.1007/s12094-025-03985-z (PMC12790528; doi:10.1007/s12094-025-03985-z)
Supplement: Supplementary file 5 — Supplementary file5 (DOCX 23 KB) [file 12094_2025_3985_MOESM5_ESM.docx]

**Supplementary 1.** Results of Joinpoint Analysis for NMSC Incidence by Sex in <45 years old in European Countries (1992–2021).

| **Location** | **MEN** | | |  | **WOMEN** | | |
| --- | --- | --- | --- | --- | --- | --- | --- |
|  | **JP** | **AAPC 1992-2021** | **APC** |  | **JP** | **AAPC 1992-2021** | **APC** |
| Austria | 5 | 0.88 (0.79; 0.98)* | 1992 - 1994: -1.29 (-1.96, -0.62)* 1994 - 2006: 0.03 (-0.01, 0.08) 2006 - 2010: -3.34 (-3.66, -3.02)* 2010 - 2013: 4.49 (3.78, 5.21)* 2013 - 2018: 5.49 (5.28, 5.70)* 2018 - 2021: 0.48 (0.19, 0.77)* |  | 4 | 1.02 (0.89; 1.15)* | 1992 - 1998: -0.56 (-0.79, -0.33)* 1998 - 2006: 0.57 (0.39, 0.74)* 2006 - 2010: -5.21 (-5.85, -4.56)* 2010 - 2018: 6.24 (6.06, 6.43)* 2018 - 2021: 0.37 (-0.21, 0.95) |
| Belgium | 4 | 0.12 (0.07; 0.16)* | 1992 - 2001: -0.13 (-0.17, -0.09)* 2001 - 2005: 0.35 (0.14, 0.56)* 2005 - 2010: -1.17 (-1.30, -1.03)* 2010 - 2015: 1.97 (1.84, 2.11)* 2015 - 2021: -0.12 (-0.19, -0.05)* |  | 3 | -0.06 (-0.09; -0.02)* | 1992 - 2005: 0.14 (0.12, 0.16)* 2005 - 2010: -2.26 (-2.37, -2.14)* 2010 - 2015: 1.65 (1.53, 1.77)* 2015 - 2021: -0.04 (-0.10, 0.02) |
| Bulgaria | 2 | 0.96 (0.28; 1.64)* | 1992 - 1995: -1.03 (-5.04, 3.15) 1995 - 1999: 6.50 (2.27, 10.90)* 1999 - 2021: 0.25 (0.07, 0.44)* |  | 3 | 1.40 (1.01; 1.80)* | 1992 - 1996: 0.45 (-0.69, 1.61) 1996 - 1999: 8.46 (4.77, 12.28)* 1999 - 2016: 0.09 (-0.04, 0.22) 2016 - 2021: 2.58 (1.79, 3.36)* |
| Croatia | 6 | 0.27 (0.26; 0.29)* | 1992 - 1996: 0.35 (0.33, 0.38)* 1996 - 1999: 1.82 (1.74, 1.90)* 1999 - 2002: 0.14 (0.06, 0.22)* 2002 - 2005: -0.19 (-0.27, -0.11)* 2005 - 2010: 0.52 (0.49, 0.55)* 2010 - 2015: -0.30 (-0.33, -0.27)* 2015 - 2021: 0.02 (0.00, 0.03)* |  | 6 | 0.01 (-0.01; 0.04) | 1992 - 1995: 0.21 (0.13, 0.30)* 1995 - 2000: -1.22 (-1.27, -1.16)* 2000 - 2005: 0.90 (0.84, 0.96)* 2005 - 2010: -0.68 (-0.74, -0.62)* 2010 - 2015: 0.32 (0.26, 0.38)* 2015 - 2018: 0.93 (0.72, 1.13)* 2018 - 2021: 0.14 (0.04, 0.25)* |
| Cyprus | 4 | 0.07 (0.05; 0.09)* | 1992 - 2001: -0.07 (-0.08, -0.05)* 2001 - 2005: 0.21 (0.13, 0.30)* 2005 - 2010: -0.53 (-0.58, -0.48)* 2010 - 2015: 0.90 (0.86, 0.95)* 2015 - 2021: -0.02 (-0.04, 0.01) |  | 2 | 0.40 (0.25; 0.54)* | 1992 - 2011: -0.07 (-0.12, -0.02)* 2011 - 2014: 3.47 (2.05, 4.92)* 2014 - 2021: 0.38 (0.21, 0.54)* |
| Czechia | 3 | -0.41 (-0.70; -0.12)* | 1992 - 2000: 2.29 (2.00, 2.57)* 2000 - 2016: 0.75 (0.65, 0.85)* 2016 - 2019: -5.02 (-7.10, -2.89)* 2019 - 2021: -12.37 (-14.68, -10.00)* |  | 2 | -0.96 (-1.22; -0.69)* | 1992 - 2000: 3.41 (3.03, 3.78)* 2000 - 2019: 0.93 (0.83, 1.02)* 2019 - 2021: -30.28 (-32.86, -27.61)* |
| Denmark | 6 | -1.26 (-1.32; -1.20)* | 1992 - 1996: 1.72 (1.61, 1.84)* 1996 - 2004: 1.10 (1.06, 1.15)* 2004 - 2008: -0.34 (-0.50, -0.18)* 2008 - 2011: -2.62 (-2.94, -2.30)* 2011 - 2015: -8.70 (-8.87, -8.53)* 2015 - 2018: -2.00 (-2.42, -1.58)* 2018 - 2021: -0.20 (-0.42, 0.02) |  | 6 | -1.53 (-1.64; -1.42)* | 1992 - 1998: 2.59 (2.48, 2.69)* 1998 - 2003: 1.39 (1.22, 1.57)* 2003 - 2006: -0.05 (-0.58, 0.49) 2006 - 2010: -5.59 (-5.86, -5.32)* 2010 - 2014: -8.06 (-8.37, -7.75)* 2014 - 2017: -3.04 (-3.77, -2.30)* 2017 - 2021: -0.25 (-0.50, 0.00) |
| Estonia | 5 | -0.26 (-0.31; -0.22)* | 1992 - 1995: 0.29 (0.11, 0.46)* 1995 - 2000: -1.27 (-1.38, -1.15)* 2000 - 2005: 0.22 (0.10, 0.34)* 2005 - 2010: 1.83 (1.71, 1.95)* 2010 - 2015: -2.65 (-2.76, -2.53)* 2015 - 2021: 0.18 (0.12, 0.25)* |  | 4 | -0.23 (-0.34; -0.11)* | 1992 - 2001: 0.02 (-0.08, 0.11) 2001 - 2005: 2.15 (1.61, 2.69)* 2005 - 2010: 0.90 (0.56, 1.23)* 2010 - 2015: -4.09 (-4.43, -3.75)* 2015 - 2021: 0.20 (-0.00, 0.40) |
| Finland | 5 | -0.02 (-0.03; -0.02)* | 1992 - 1995: 0.05 (0.03, 0.06)* 1995 - 2000: -0.07 (-0.08, -0.06)* 2000 - 2005: 0.06 (0.05, 0.07)* 2005 - 2010: -0.29 (-0.30, -0.28)* 2010 - 2015: 0.14 (0.13, 0.15)* 2015 - 2021: -0.01 (-0.01, -0.00)* |  | 5 | -0.01 (-0.05; 0.03) | 1992 - 1995: -0.12 (-0.26, 0.02) 1995 - 2000: 0.22 (0.13, 0.31)* 2000 - 2005: 0.04 (-0.05, 0.13) 2005 - 2014: -0.22 (-0.25, -0.19)* 2014 - 2018: -0.06 (-0.21, 0.10) 2018 - 2021: 0.33 (0.17, 0.48)* |
| France | 5 | -0.50 (-0.55; -0.44)* | 1992 - 1995: 0.35 (0.20, 0.51)* 1995 - 2000: 1.42 (1.32, 1.51)* 2000 - 2003: -1.46 (-1.75, -1.17)* 2003 - 2006: -2.41 (-2.70, -2.11)* 2006 - 2009: -3.41 (-3.72, -3.10)* 2009 - 2021: -0.04 (-0.06, -0.02)* |  | 5 | -1.01 (-1.08; -0.95)* | 1992 - 1996: 0.01 (-0.14, 0.16) 1996 - 2000: -0.98 (-1.22, -0.73)* 2000 - 2009: -3.00 (-3.06, -2.95)* 2009 - 2015: -0.12 (-0.25, 0.01) 2015 - 2019: 0.89 (0.60, 1.18)* 2019 - 2021: -0.54 (-1.11, 0.04) |
| Germany | 4 | 0.97 (0.70; 1.24)* | 1992 - 2005: 0.92 (0.80, 1.05)* 2005 - 2010: -1.29 (-2.03, -0.55)* 2010 - 2015: 10.62 (9.82, 11.43)* 2015 - 2019: -7.04 (-8.06, -6.01)* 2019 - 2021: 0.68 (-1.59, 3.00) |  | 4 | 0.84 (0.45; 1.23)* | 1992 - 2004: 1.50 (1.28, 1.72)* 2004 - 2010: -0.65 (-1.43, 0.13) 2010 - 2015: 13.28 (12.11, 14.45)* 2015 - 2019: -12.52 (-13.93, -11.09)* 2019 - 2021: 0.72 (-2.83, 4.41) |
| Greece | 4 | -0.00 (-0.01; 0.00) | 1992 - 1995: 0.03 (-0.01, 0.07) 1995 - 2000: -0.32 (-0.35, -0.30)* 2000 - 2005: 0.29 (0.27, 0.31)* 2005 - 2010: 0.10 (0.07, 0.12)* 2010 - 2021: -0.05 (-0.05, -0.04)* |  | 6 | 0.01 (-0.00; 0.02) | 1992 - 1995: -0.23 (-0.27, -0.20)* 1995 - 2000: 0.12 (0.10, 0.14)* 2000 - 2005: 0.26 (0.24, 0.28)* 2005 - 2010: -0.24 (-0.26, -0.22)* 2010 - 2015: -0.15 (-0.17, -0.13)* 2015 - 2019: 0.21 (0.18, 0.25)* 2019 - 2021: 0.07 (-0.00, 0.14) |
| Hungary | 6 | -0.35 (-0.37; -0.33)* | 1992 - 1994: -2.61 (-2.71, -2.51)* 1994 - 1997: -1.04 (-1.15, -0.93)* 1997 - 2000: -0.26 (-0.37, -0.15)* 2000 - 2005: 0.18 (0.14, 0.22)* 2005 - 2015: -0.04 (-0.05, -0.03)* 2015 - 2018: -0.40 (-0.51, -0.29)* 2018 - 2021: -0.06 (-0.12, -0.01)* |  | 2 | -0.24 (-0.26; -0.22)* | 1992 - 1995: 0.28 (0.14, 0.43)* 1995 - 2000: -1.75 (-1.84, -1.65)* 2000 - 2021: 0.05 (0.04, 0.06)* |
| Ireland | 4 | -0.95 (-1.00; -0.90)* | 1992 - 2002: 0.65 (0.61, 0.68)* 2002 - 2006: -0.24 (-0.45, -0.02)* 2006 - 2010: -4.64 (-4.85, -4.44)* 2010 - 2014: -3.47 (-3.68, -3.25)* 2014 - 2021: 0.01 (-0.06, 0.07) |  | 5 | -0.78 (-0.92; -0.64)* | 1992 - 2000: 0.86 (0.76, 0.97)* 2000 - 2003: 1.63 (0.73, 2.54)* 2003 - 2006: -0.02 (-0.86, 0.83) 2006 - 2010: -5.59 (-6.00, -5.18)* 2010 - 2014: -2.88 (-3.32, -2.43)* 2014 - 2021: 0.04 (-0.08, 0.17) |
| Italy | 2 | -0.04 (-0.16; 0.08) | 1992 - 2015: 1.13 (1.10, 1.15)* 2015 - 2019: -6.68 (-7.24, -6.12)* 2019 - 2021: 0.35 (-0.98, 1.70) |  | 3 | -0.15 (-0.35; 0.05) | 1992 - 2009: 1.71 (1.65, 1.78)* 2009 - 2015: 1.15 (0.78, 1.52)* 2015 - 2019: -9.60 (-10.44, -8.75)* 2019 - 2021: 0.12 (-2.03, 2.32) |
| Latvia | 6 | 0.67 (0.65; 0.68)* | 1992 - 2000: 0.03 (0.02, 0.04)* 2000 - 2005: -0.73 (-0.76, -0.71)* 2005 - 2008: 1.22 (1.14, 1.30)* 2008 - 2011: 2.42 (2.34, 2.50)* 2011 - 2014: 3.69 (3.61, 3.77)* 2014 - 2017: 0.39 (0.31, 0.47)* 2017 - 2021: -0.05 (-0.08, -0.03)* |  | 4 | 0.67 (0.64; 0.70)* | 1992 - 2005: 0.03 (0.02, 0.03)* 2005 - 2008: 0.97 (0.81, 1.14)* 2008 - 2011: 1.83 (1.66, 2.00)* 2011 - 2014: 3.60 (3.43, 3.77)* 2014 - 2021: 0.01 (-0.01, 0.04) |
| Lithuania | 4 | -0.00 (-0.03; 0.02) | 1992 - 2001: 0.00 (-0.01, 0.02) 2001 - 2005: 0.59 (0.49, 0.69)* 2005 - 2010: 1.11 (1.04, 1.18)* 2010 - 2015: -1.59 (-1.66, -1.52)* 2015 - 2021: 0.00 (-0.04, 0.04) |  | 4 | -0.22 (-0.30; -0.13)* | 1992 - 2000: -0.03 (-0.09, 0.02) 2000 - 2008: 1.30 (1.23, 1.37)* 2008 - 2011: -0.17 (-0.72, 0.38) 2011 - 2014: -4.76 (-5.33, -4.20)* 2014 - 2021: -0.17 (-0.26, -0.08)* |
| Luxembourg | 4 | -0.00 (-0.01; 0.01) | 1992 - 1995: -0.02 (-0.06, 0.02) 1995 - 2000: -0.10 (-0.12, -0.07)* 2000 - 2005: 0.32 (0.29, 0.34)* 2005 - 2010: -0.13 (-0.15, -0.11)* 2010 - 2021: -0.04 (-0.05, -0.04)* |  | 6 | -0.02 (-0.02; -0.01)* | 1992 - 1995: -0.13 (-0.15, -0.10)* 1995 - 1999: 0.20 (0.18, 0.23)* 1999 - 2005: 0.09 (0.07, 0.10)* 2005 - 2010: -0.07 (-0.08, -0.05)* 2010 - 2015: -0.28 (-0.30, -0.27)* 2015 - 2019: 0.20 (0.18, 0.22)* 2019 - 2021: -0.25 (-0.29, -0.21)* |
| Malta | 4 | -0.59 (-0.71; -0.47)* | 1992 - 1994: 1.58 (0.76, 2.42)* 1994 - 2012: 0.03 (0.00, 0.06)* 2012 - 2015: -1.05 (-1.85, -0.25)* 2015 - 2019: -4.49 (-4.88, -4.09)* 2019 - 2021: 0.39 (-0.47, 1.26) |  | 5 | -0.91 (-0.99; -0.82)* | 1992 - 1994: 1.50 (0.91, 2.09)* 1994 - 2001: 0.03 (-0.07, 0.12) 2001 - 2011: 0.67 (0.62, 0.72)* 2011 - 2015: -3.54 (-3.82, -3.26)* 2015 - 2018: -6.08 (-6.66, -5.50)* 2018 - 2021: -1.01 (-1.33, -0.68)* |
| Netherlands | 3 | 0.56 (0.54; 0.57)* | 1992 - 1995: 1.95 (1.88, 2.03)* 1995 - 1999: 2.19 (2.12, 2.27)* 1999 - 2003: 0.41 (0.34, 0.48)* 2003 - 2021: -0.00 (-0.01, 0.00) |  |  | 0.37 (0.33; 0.42)* | 1992 - 1995: 1.11 (0.95, 1.28)* 1995 - 1999: 2.00 (1.83, 2.16)* 1999 - 2010: 0.15 (0.13, 0.17)* 2010 - 2015: -0.39 (-0.49, -0.29)* 2015 - 2019: 0.23 (0.06, 0.39)* 2019 - 2021: -0.51 (-0.84, -0.17)* |
| Poland | 4 | 0.91 (0.88; 0.94)* | 1992 - 2003: -0.07 (-0.08, -0.05)* 2003 - 2006: 0.87 (0.66, 1.07)* 2006 - 2009: 7.79 (7.59, 7.99)* 2009 - 2012: 0.59 (0.41, 0.76)* 2012 - 2021: 0.01 (-0.01, 0.02) |  | 5 | 1.03 (1.01; 1.05)* | 1992 - 2003: -0.04 (-0.05, -0.03)* 2003 - 2006: 1.11 (0.99, 1.22)* 2006 - 2009: 8.52 (8.41, 8.64)* 2009 - 2012: 0.75 (0.65, 0.85)* 2012 - 2021: -0.01 (-0.01, 0.00) |
| Portugal | 5 | 1.14 (1.06; 1.22)* | 1992 - 1995: 0.12 (-0.21, 0.45) 1995 - 2003: 1.21 (1.13, 1.30)* 2003 - 2010: 0.90 (0.81, 1.00)* 2010 - 2014: 3.93 (3.64, 4.21)* 2014 - 2017: 0.54 (0.00, 1.08)* 2017 - 2021: -0.08 (-0.26, 0.09) |  | 4 | 1.15 (1.09; 1.20)* | 1992 - 1996: -0.10 (-0.22, 0.02) 1996 - 1999: 2.39 (2.01, 2.76)* 1999 - 2006: 0.28 (0.22, 0.34)* 2006 - 2010: 2.47 (2.30, 2.64)* 2010 - 2014: 3.67 (3.50, 3.83)* 2014 - 2017: 0.34 (0.04, 0.65)* 2017 - 2021: -0.20 (-0.30, -0.10)* |
| Romania | 5 | 0.13 (0.13; 0.14)* | 1992 - 1994: 1.31 (1.26, 1.35)* 1994 - 1997: 0.55 (0.51, 0.60)* 1997 - 2000: 0.21 (0.17, 0.25)* 2000 - 2005: -0.38 (-0.40, -0.37)* 2005 - 2015: 0.09 (0.09, 0.09)* 2015 - 2021: 0.01 (0.00, 0.02)* |  | 6 | 0.40 (0.37; 0.43)* | 1992 - 1994: 1.98 (1.73, 2.23)* 1994 - 1998: 1.36 (1.24, 1.48)* 1998 - 2001: 0.49 (0.26, 0.73)* 2001 - 2013: -0.02 (-0.04, -0.01)* 2013 - 2021: 0.13 (0.10, 0.16)* |
| Slovakia | 4 | -0.87 (-1.00; -0.74)* | 1992 - 2008: -0.20 (-0.23, -0.17)* 2008 - 2011: -1.28 (-1.99, -0.56)* 2011 - 2014: -5.19 (-5.90, -4.48)* 2014 - 2017: -0.82 (-1.57, -0.06)* 2017 - 2021: 0.05 (-0.19, 0.29) |  | 4 | -0.49 (-0.70; -0.28)* | 1992 - 2011: 0.34 (0.28, 0.40)* 2011 - 2014: -6.31 (-8.17, -4.41)* 2014 - 2021: -0.15 (-0.43, 0.13) |
| Slovenia | 4 | 1.03 (0.95; 1.11)* | 1992 - 1999: 5.25 (5.16, 5.35)* 1999 - 2002: 1.91 (1.28, 2.55)* 2002 - 2012: 0.94 (0.89, 1.00)* 2012 - 2019: 0.50 (0.40, 0.60)* 2019 - 2021: -11.66 (-12.23, -11.09)* |  | 2 | 1.45 (1.36; 1.53)* | 1992 - 1997: 7.39 (7.23, 7.56)* 1997 - 2000: 5.14 (4.54, 5.75)* 2000 - 2010: 1.40 (1.35, 1.45)* 2010 - 2015: 0.32 (0.15, 0.49)* 2015 - 2019: 1.44 (1.17, 1.71)* 2019 - 2021: -14.03 (-14.53, -13.52)* |
| Spain | 3 | -0.87 (-1.09; -0.66)* | 1992 - 2006: 0.69 (0.60, 0.78)* 2006 - 2011: -3.50 (-4.02, -2.98)* 2011 - 2014: -7.16 (-8.89, -5.39)* 2014 - 2021: 0.72 (0.44, 1.00)* |  | 5 | -1.03 (-1.26; -0.80)* | 1992 - 2006: 1.00 (0.91, 1.10)* 2006 - 2011: -4.66 (-5.21, -4.11)* 2011 - 2014: -8.42 (-10.29, -6.51)* 2014 - 2021: 0.89 (0.58, 1.20)* |
| Sweden | 4 | -0.37 (-0.48; -0.26)* | 1992 - 1994: -17.31 (-17.92, -16.70)* 1994 - 1997: -2.33 (-3.19, -1.47)* 1997 - 2012: 0.11 (0.06, 0.15)* 2012 - 2019: 1.04 (0.89, 1.18)* 2019 - 2021: 13.64 (12.80, 14.49)* |  | 3 | -0.47 (-0.80; -0.13)* | 1992 - 1994: -22.45 (-24.87, -19.95)* 1994 - 2013: 0.20 (0.08, 0.32)* 2013 - 2019: 2.08 (1.28, 2.89)* 2019 - 2021: 11.13 (7.63, 14.74)* |
| United Kingdom | 5 | -0.13 (-0.19; -0.07)* | 1992 - 1994: 3.16 (2.71, 3.60)* 1994 - 2006: 0.15 (0.12, 0.18)* 2006 - 2009: 2.60 (2.20, 3.00)* 2009 - 2015: -0.26 (-0.35, -0.17)* 2015 - 2019: -4.54 (-4.74, -4.35)* 2019 - 2021: 0.29 (-0.14, 0.71) |  | 3 | -0.05 (-0.10; 0.01) | 1992 - 1994: 3.51 (3.12, 3.91)* 1994 - 2006: 0.26 (0.23, 0.28)* 2006 - 2009: 4.44 (4.09, 4.79)* 2009 - 2015: 0.01 (-0.06, 0.09) 2015 - 2019: -5.80 (-5.96, -5.63)* 2019 - 2021: -0.27 (-0.63, 0.10) |
| CENTRAL/  EASTERN | 6 | 0.20 (0.14; 0.26)* | 1992 - 1995: -0.15 (-0.36, 0.05) 1995 - 1999: 1.39 (1.18, 1.60)* 1999 - 2005: 0.68 (0.59, 0.78)* 2005 - 2009: 1.73 (1.52, 1.94)* 2009 - 2016: 0.25 (0.19, 0.32)* 2016 - 2019: -1.48 (-1.86, -1.10)* 2019 - 2021: -3.70 (-4.10, -3.31)* |  | 5 | 0.26 (0.13; 0.39)* | 1992 - 2010: 1.28 (1.22, 1.33)* 2010 - 2019: 0.38 (0.20, 0.56)* 2019 - 2021: -8.99 (-10.58, -7.38)* |
| NORTHERN | 5 | -0.19 (-0.23; -0.16)* | 1992 - 1995: 1.17 (1.04, 1.30)* 1995 - 2005: 0.34 (0.31, 0.36)* 2005 - 2010: 1.07 (0.99, 1.15)* 2010 - 2015: -1.12 (-1.20, -1.04)* 2015 - 2019: -3.44 (-3.56, -3.31)* 2019 - 2021: 0.92 (0.65, 1.20)* |  | 2 | -0.13 (-0.18; -0.09)* | 1992 - 1994: 1.78 (1.47, 2.09)* 1994 - 2006: 0.57 (0.55, 0.59)* 2006 - 2009: 2.26 (1.98, 2.54)* 2009 - 2015: -0.68 (-0.74, -0.62)* 2015 - 2019: -4.36 (-4.50, -4.22)* 2019 - 2021: 0.49 (0.18, 0.79)* |
| SOUTHERN | 5 | -0.27 (-0.38; -0.16)* | 1992 - 2000: 0.90 (0.82, 0.99)* 2000 - 2005: 1.42 (1.20, 1.65)* 2005 - 2009: -0.26 (-0.59, 0.07) 2009 - 2016: -1.59 (-1.71, -1.48)* 2016 - 2019: -3.25 (-3.99, -2.51)* 2019 - 2021: 0.05 (-0.75, 0.87) |  | 5 | -0.36 (-0.57; -0.15)* | 1992 - 2006: 1.44 (1.37, 1.51)* 2006 - 2010: -0.89 (-1.53, -0.23)* 2010 - 2016: -1.93 (-2.23, -1.62)* 2016 - 2019: -5.33 (-6.79, -3.85)* 2019 - 2021: 0.50 (-1.15, 2.18) |
| WESTERN | 5 | 0.19 (0.10; 0.29)* | 1992 - 2000: 0.76 (0.68, 0.84)* 2000 - 2005: -0.57 (-0.79, -0.34)* 2005 - 2010: -1.60 (-1.83, -1.37)* 2010 - 2015: 4.50 (4.25, 4.75)* 2015 - 2019: -2.89 (-3.25, -2.52)* 2019 - 2021: 0.08 (-0.69, 0.84) |  | 4 | -0.17 (-0.33; -0.02)* | 1992 - 2000: 0.15 (0.01, 0.29)* 2000 - 2010: -1.32 (-1.44, -1.19)* 2010 - 2015: 5.60 (5.15, 6.06)* 2015 - 2019: -4.96 (-5.60, -4.31)* 2019 - 2021: 0.03 (-1.38, 1.46) |
| UE28 | 5 | 0.00 (-0.06; 0.07) | 1992 - 2002: 0.94 (0.90, 0.97)* 2002 - 2006: 0.52 (0.30, 0.74)* 2006 - 2011: -0.27 (-0.40, -0.13)* 2011 - 2015: 0.56 (0.34, 0.78)* 2015 - 2019: -2.85 (-3.08, -2.63)* 2019 - 2021: -0.29 (-0.77, 0.19) |  | 4 | -0.15 (-0.25; -0.05)* | 1992 - 2005: 0.83 (0.78, 0.87)* 2005 - 2011: -0.11 (-0.30, 0.07) 2011 - 2015: 1.05 (0.64, 1.46)* 2015 - 2019: -4.03 (-4.44, -3.63)* 2019 - 2021: -1.05 (-1.94, -0.16)* |

AAPC: Anual Average percentage change. JP: Joinpoint. APC: Annual Percentage Change and 95% confidence interval. * = p<0.05

Western countries: green, Southern countries: red, Northern countries: blue, Central and Eastern countries: yellow.
