# Supplementary material for: Incidence and mortality of nonmelanoma skin cancer in Europe: current trends and challenges
Source: Clin Transl Oncol. 2025 Jul 11;28(1):302–19. doi: 10.1007/s12094-025-03985-z (PMC12790528; doi:10.1007/s12094-025-03985-z)
Supplement: Supplementary file 6 — Supplementary file6 (DOCX 22 KB) [file 12094_2025_3985_MOESM6_ESM.docx]

**Supplementary table 2.** Results of Joinpoint Analysis for NMSC Incidence by Sex in 45-74 years old in European Countries (1992–2021).

| **Location** | **MEN** | | |  | **WOMEN** | | |
| --- | --- | --- | --- | --- | --- | --- | --- |
|  | **JP** | **AAPC 1992-2021** | **APC** |  | **JP** | **AAPC 1992-2021** | **APC** |
| Austria | 5 | 0.76 (0.59; 0.93)* | 1992 - 1995: -1.62 (-2.29, -0.93)* 1995 - 2006: 0.20 (0.10, 0.31)* 2006 - 2010: -3.26 (-3.87, -2.64)* 2010 - 2015: 0.37 (-0.02, 0.76) 2015 - 2018: 11.01 (9.75, 12.28)* 2018 - 2021: 1.56 (1.05, 2.07)* |  | 5 | 0.52 (0.36; 0.67)* | 1992 - 1995: -1.64 (-2.25, -1.02)* 1995 - 2006: 0.30 (0.20, 0.39)* 2006 - 2010: -4.15 (-4.75, -3.54)* 2010 - 2015: -0.28 (-0.67, 0.12) 2015 - 2019: 9.08 (8.46, 9.70)* 2019 - 2021: 0.10 (-0.91, 1.13) |
| Belgium | 3 | 0.18 (0.13; 0.23)* | 1992 - 2006: 0.05 (0.02, 0.08)* 2006 - 2010: -2.25 (-2.55, -1.96)* 2010 - 2015: 2.97 (2.79, 3.16)* 2015 - 2021: -0.18 (-0.27, -0.09)* |  | 3 | 0.23 (0.15; 0.31)* | 1992 - 2005: 0.14 (0.09, 0.18)* 2005 - 2010: -0.98 (-1.24, -0.71)* 2010 - 2014: 2.00 (1.58, 2.43)* 2014 - 2021: 0.26 (0.16, 0.37)* |
| Bulgaria | 3 | 0.57 (0.46; 0.69)* | 1992 - 1999: 4.06 (3.92, 4.20)* 1999 - 2016: -0.03 (-0.07, 0.01) 2016 - 2019: 3.20 (2.27, 4.14)* 2019 - 2021: -9.63 (-10.48, -8.77)* |  | 3 | 1.05 (0.99; 1.10)* | 1992 - 1999: 3.87 (3.80, 3.94)* 1999 - 2016: 0.00 (-0.02, 0.02) 2016 - 2019: 2.38 (1.90, 2.86)* 2019 - 2021: -1.73 (-2.19, -1.28)* |
| Croatia | 4 | 0.21 (0.18; 0.23)* | 1992 - 1995: -0.22 (-0.32, -0.13)* 1995 - 2000: 0.84 (0.78, 0.90)* 2000 - 2015: 0.05 (0.04, 0.06)* 2015 - 2019: 0.45 (0.37, 0.53)* 2019 - 2021: -0.04 (-0.20, 0.12) |  | 6 | 0.09 (0.07; 0.11)* | 1992 - 1995: 0.14 (0.06, 0.22)* 1995 - 2000: -0.87 (-0.92, -0.82)* 2000 - 2005: 0.78 (0.73, 0.82)* 2005 - 2010: -0.59 (-0.64, -0.54)* 2010 - 2015: 0.46 (0.41, 0.51)* 2015 - 2018: 0.97 (0.82, 1.13)* 2018 - 2021: 0.14 (0.07, 0.21)* |
| Cyprus | 3 | 0.29 (0.22; 0.36)* | 1992 - 2011: 0.03 (0.01, 0.04)* 2011 - 2014: 2.22 (1.72, 2.73)* 2014 - 2017: 0.41 (-0.05, 0.87) 2017 - 2021: 0.03 (-0.10, 0.17) |  | 6 | 0.00 (-0.00; 0.01) | 1992 - 1995: -0.11 (-0.14, -0.09)* 1995 - 2000: 0.03 (0.02, 0.04)* 2000 - 2005: -0.16 (-0.17, -0.15)* 2005 - 2010: 0.08 (0.07, 0.09)* 2010 - 2014: -0.05 (-0.07, -0.03)* 2014 - 2019: 0.19 (0.18, 0.20)* 2019 - 2021: -0.04 (-0.07, -0.01)* |
| Czechia | 3 | -1.26 (-1.45; -1.06)* | 1992 - 1999: 4.05 (3.78, 4.32)* 1999 - 2016: 1.09 (1.03, 1.15)* 2016 - 2019: -10.98 (-12.24, -9.71)* 2019 - 2021: -21.31 (-22.75, -19.84)* |  | 2 | -1.28 (-1.45; -1.12)* | 1992 - 1999: 4.26 (3.96, 4.57)* 1999 - 2019: 1.14 (1.09, 1.19)* 2019 - 2021: -36.03 (-37.47, -34.55)* |
| Denmark | 4 | -0.85 (-0.98; -0.72)* | 1992 - 2002: 1.54 (1.45, 1.62)* 2002 - 2008: 0.22 (-0.01, 0.44) 2008 - 2011: -1.79 (-2.73, -0.84)* 2011 - 2015: -8.19 (-8.66, -7.71)* 2015 - 2021: -0.28 (-0.46, -0.09)* |  | 6 | -1.31 (-1.42; -1.19)* | 1992 - 1999: 2.41 (2.32, 2.50)* 1999 - 2003: 1.55 (1.24, 1.85)* 2003 - 2006: 0.27 (-0.31, 0.86) 2006 - 2011: -5.69 (-5.87, -5.51)* 2011 - 2014: -8.11 (-8.72, -7.49)* 2014 - 2017: -2.38 (-3.07, -1.69)* 2017 - 2021: 0.03 (-0.19, 0.25) |
| Estonia | 3 | -0.33 (-0.38; -0.29)* | 1992 - 2000: 0.26 (0.18, 0.33)* 2000 - 2010: 1.16 (1.11, 1.22)* 2010 - 2015: -4.70 (-4.89, -4.51)* 2015 - 2021: 0.12 (0.01, 0.23)* |  | 6 | -0.40 (-0.43; -0.38)* | 1992 - 2000: 0.65 (0.63, 0.66)* 2000 - 2004: 2.46 (2.39, 2.52)* 2004 - 2008: 0.96 (0.89, 1.02)* 2008 - 2011: -0.65 (-0.78, -0.52)* 2011 - 2014: -8.17 (-8.30, -8.04)* 2014 - 2017: -1.19 (-1.35, -1.04)* 2017 - 2021: 0.20 (0.15, 0.25)* |
| Finland | 5 | -0.03 (-0.03; -0.03)* | 1992 - 1995: -0.02 (-0.03, -0.00)* 1995 - 2000: -0.18 (-0.19, -0.17)* 2000 - 2005: 0.15 (0.15, 0.16)* 2005 - 2010: 0.03 (0.02, 0.03)* 2010 - 2015: -0.16 (-0.16, -0.15)* 2015 - 2021: 0.00 (-0.00, 0.01) |  | 4 | 0.02 (-0.03; 0.07) | 1992 - 1994: -0.44 (-0.95, 0.08) 1994 - 2004: 0.18 (0.13, 0.22)* 2004 - 2013: -0.22 (-0.27, -0.17)* 2013 - 2018: 0.04 (-0.10, 0.18) 2018 - 2021: 0.54 (0.32, 0.76)* |
| France | 5 | -0.86 (-1.03; -0.69)* | 1992 - 2000: 1.71 (1.61, 1.81)* 2000 - 2003: -2.18 (-3.00, -1.35)* 2003 - 2006: -3.92 (-4.76, -3.08)* 2006 - 2009: -5.96 (-6.83, -5.08)* 2009 - 2012: -0.58 (-1.53, 0.38) 2012 - 2021: 0.02 (-0.06, 0.10) |  | 5 | -1.10 (-1.22; -0.98)* | 1992 - 1997: 0.20 (-0.01, 0.41) 1997 - 2001: -1.32 (-1.78, -0.87)* 2001 - 2005: -4.98 (-5.44, -4.52)* 2005 - 2009: -2.58 (-3.08, -2.08)* 2009 - 2014: -0.41 (-0.73, -0.09)* 2014 - 2021: 0.74 (0.62, 0.87)* |
| Germany | 4 | 0.84 (0.39; 1.30)* | 1992 - 2004: 1.45 (1.15, 1.76)* 2004 - 2010: -0.79 (-1.77, 0.20) 2010 - 2015: 17.02 (15.66, 18.39)* 2015 - 2019: -15.59 (-17.11, -14.05)* 2019 - 2021: 0.50 (-3.54, 4.70) |  | 4 | 0.95 (0.45; 1.45)* | 1992 - 2004: 1.68 (1.36, 2.00)* 2004 - 2010: -0.65 (-1.73, 0.44) 2010 - 2015: 17.76 (16.28, 19.26)* 2015 - 2019: -16.37 (-17.99, -14.72)* 2019 - 2021: 0.48 (-3.97, 5.13) |
| Greece | 3 | 0.03 (0.02; 0.03)* | 1992 - 1995: 0.16 (0.14, 0.19)* 1995 - 2000: -0.06 (-0.08, -0.05)* 2000 - 2009: 0.08 (0.07, 0.08)* 2009 - 2021: -0.01 (-0.01, -0.01)* |  | 5 | 0.03 (-0.01; 0.06) | 1992 - 1995: -0.20 (-0.37, -0.03)* 1995 - 2000: 0.37 (0.27, 0.48)* 2000 - 2005: 0.10 (-0.00, 0.20) 2005 - 2010: -0.23 (-0.33, -0.12)* 2010 - 2018: -0.11 (-0.15, -0.07)* 2018 - 2021: 0.36 (0.19, 0.52)* |
| Hungary | 2 | -0.40 (-0.45; -0.36)* | 1992 - 1995: -0.04 (-0.34, 0.27) 1995 - 2000: -2.41 (-2.60, -2.22)* 2000 - 2021: 0.03 (0.02, 0.05)* |  | 3 | -0.34 (-0.40; -0.27)* | 1992 - 1996: -0.42 (-0.60, -0.23)* 1996 - 1999: -3.19 (-3.76, -2.61)* 1999 - 2015: 0.00 (-0.02, 0.03) 2015 - 2021: 0.27 (0.17, 0.37)* |
| Ireland | 4 | -1.69 (-1.76; -1.62)* | 1992 - 2003: 0.91 (0.87, 0.95)* 2003 - 2006: -0.41 (-0.93, 0.11) 2006 - 2010: -7.43 (-7.68, -7.18)* 2010 - 2014: -6.47 (-6.74, -6.19)* 2014 - 2021: -0.08 (-0.16, -0.00)* |  | 5 | -1.91 (-2.13; -1.69)* | 1992 - 2000: 1.11 (0.95, 1.27)* 2000 - 2003: 3.36 (1.96, 4.79)* 2003 - 2006: 0.23 (-1.04, 1.53) 2006 - 2010: -11.83 (-12.44, -11.21)* 2010 - 2014: -6.37 (-7.12, -5.62)* 2014 - 2021: 0.18 (-0.03, 0.39) |
| Italy | 3 | -0.15 (-0.21; -0.08)* | 1992 - 2009: 0.90 (0.87, 0.92)* 2009 - 2015: 0.04 (-0.10, 0.18) 2015 - 2019: -4.96 (-5.27, -4.65)* 2019 - 2021: 0.34 (-0.32, 1.01) |  | 3 | 0.23 (0.14; 0.32)* | 1992 - 2009: 1.23 (1.20, 1.27)* 2009 - 2015: 0.37 (0.18, 0.57)* 2015 - 2019: -4.31 (-4.73, -3.89)* 2019 - 2021: 0.63 (-0.26, 1.53) |
| Latvia | 4 | 0.21 (0.19; 0.24)* | 1992 - 2000: 0.04 (0.01, 0.06)* 2000 - 2005: -0.35 (-0.41, -0.29)* 2005 - 2011: 0.90 (0.85, 0.94)* 2011 - 2014: 0.48 (0.28, 0.69)* 2014 - 2021: 0.11 (0.09, 0.14)* |  | 4 | 0.34 (0.32; 0.37)* | 1992 - 2001: -0.01 (-0.02, 0.01) 2001 - 2005: 0.39 (0.29, 0.49)* 2005 - 2010: 1.19 (1.13, 1.26)* 2010 - 2014: 0.84 (0.74, 0.94)* 2014 - 2021: -0.11 (-0.14, -0.09)* |
| Lithuania | 4 | -0.03 (-0.08; 0.02) | 1992 - 2001: 0.05 (0.01, 0.08)* 2001 - 2004: 2.18 (1.78, 2.58)* 2004 - 2010: 1.24 (1.15, 1.32)* 2010 - 2015: -2.82 (-2.94, -2.70)* 2015 - 2021: -0.12 (-0.18, -0.05)* |  | 3 | -0.18 (-0.27; -0.08)* | 1992 - 2000: -0.01 (-0.15, 0.14) 2000 - 2010: 2.29 (2.18, 2.41)* 2010 - 2015: -5.49 (-5.87, -5.11)* 2015 - 2021: 0.08 (-0.15, 0.31) |
| Luxembourg | 6 | 0.01 (0.01; 0.02)* | 1992 - 1995: 0.04 (0.02, 0.07)* 1995 - 2000: -0.22 (-0.24, -0.20)* 2000 - 2005: 0.36 (0.34, 0.37)* 2005 - 2010: -0.19 (-0.21, -0.18)* 2010 - 2015: 0.21 (0.19, 0.22)* 2015 - 2019: -0.13 (-0.15, -0.11)* 2019 - 2021: 0.01 (-0.03, 0.05) |  | 4 | 0.02 (-0.02; 0.06) | 1992 - 1996: -0.08 (-0.20, 0.05) 1996 - 2005: 0.18 (0.14, 0.22)* 2005 - 2015: -0.22 (-0.25, -0.19)* 2015 - 2018: 0.07 (-0.27, 0.41) 2018 - 2021: 0.42 (0.26, 0.58)* |
| Malta | 4 | -0.92 (-1.02; -0.82)* | 1992 - 2001: 0.07 (-0.02, 0.16) 2001 - 2011: 0.72 (0.65, 0.80)* 2011 - 2015: -3.26 (-3.63, -2.89)* 2015 - 2018: -5.97 (-6.68, -5.24)* 2018 - 2021: -0.98 (-1.38, -0.59)* |  | 5 | -0.35 (-0.41; -0.30)* | 1992 - 1994: 2.19 (1.69, 2.69)* 1994 - 2001: 0.11 (0.03, 0.19)* 2001 - 2010: 1.51 (1.46, 1.55)* 2010 - 2015: -0.89 (-1.01, -0.78)* 2015 - 2019: -6.59 (-6.77, -6.41)* 2019 - 2021: 1.44 (1.03, 1.84)* |
| Netherlands | 3 | 0.95 (0.85; 1.05)* | 1992 - 1999: 4.06 (3.89, 4.24)* 1999 - 2003: 0.88 (0.33, 1.44)* 2003 - 2019: -0.03 (-0.07, 0.01) 2019 - 2021: -1.70 (-2.56, -0.83)* |  | 6 | 0.52 (0.49; 0.55)* | 1992 - 1995: 2.02 (1.90, 2.13)* 1995 - 1999: 3.27 (3.16, 3.39)* 1999 - 2002: 0.35 (0.15, 0.56)* 2002 - 2007: -0.18 (-0.24, -0.12)* 2007 - 2015: -0.37 (-0.39, -0.34)* 2015 - 2019: 0.10 (0.01, 0.19)* 2019 - 2021: -0.67 (-0.84, -0.49)* |
| Poland | 4 | 1.04 (0.96; 1.12)* | 1992 - 2003: -0.06 (-0.10, -0.03)* 2003 - 2006: 1.32 (0.80, 1.84)* 2006 - 2009: 8.97 (8.46, 9.48)* 2009 - 2012: 0.95 (0.51, 1.38)* 2012 - 2021: -0.19 (-0.22, -0.15)* |  | 3 | 1.19 (1.14; 1.25)* | 1992 - 2003: -0.08 (-0.10, -0.05)* 2003 - 2006: 1.21 (0.81, 1.60)* 2006 - 2009: 10.28 (9.89, 10.67)* 2009 - 2021: 0.19 (0.18, 0.21)* |
| Portugal | 4 | 2.43 (2.23; 2.64)* | 1992 - 1996: 0.39 (-0.30, 1.09) 1996 - 2010: 2.73 (2.62, 2.83)* 2010 - 2014: 7.14 (6.33, 7.96)* 2014 - 2017: 1.04 (-0.33, 2.43) 2017 - 2021: -0.05 (-0.46, 0.37) |  | 5 | 1.21 (1.14; 1.29)* | 1992 - 1996: 0.14 (-0.02, 0.31) 1996 - 1999: 2.54 (2.04, 3.04)* 1999 - 2007: 0.74 (0.68, 0.81)* 2007 - 2011: 2.04 (1.81, 2.26)* 2011 - 2014: 4.04 (3.62, 4.46)* 2014 - 2021: 0.15 (0.10, 0.20)* |
| Romania | 1 | 1.09 (1.06; 1.11)* | 1992 - 1999: 4.45 (4.36, 4.54)* 1999 - 2021: 0.04 (0.03, 0.05)* |  | 4 | 1.08 (1.04; 1.13)* | 1992 - 1995: 3.93 (3.71, 4.14)* 1995 - 1999: 4.55 (4.36, 4.74)* 1999 - 2002: 0.49 (0.14, 0.83)* 2002 - 2011: -0.11 (-0.15, -0.07)* 2011 - 2021: 0.15 (0.12, 0.17)* |
| Slovakia | 3 | -0.72 (-0.79; -0.65)* | 1992 - 2005: 0.42 (0.37, 0.47)* 2005 - 2010: 2.67 (2.38, 2.97)* 2010 - 2015: -7.77 (-8.03, -7.50)* 2015 - 2021: 0.14 (-0.02, 0.29) |  | 3 | -0.61 (-0.69; -0.54)* | 1992 - 2005: 0.28 (0.23, 0.33)* 2005 - 2010: 3.13 (2.84, 3.41)* 2010 - 2015: -7.44 (-7.70, -7.18)* 2015 - 2021: 0.29 (0.14, 0.45)* |
| Slovenia | 2 | 1.18 (1.09; 1.27)* | 1992 - 2000: 5.22 (5.03, 5.41)* 2000 - 2019: 0.67 (0.63, 0.71)* 2019 - 2021: -9.29 (-10.29, -8.28)* |  | 2 | 1.00 (0.92; 1.09)* | 1992 - 2000: 5.11 (4.96, 5.27)* 2000 - 2019: 0.84 (0.80, 0.87)* 2019 - 2021: -12.53 (-13.46, -11.58)* |
| Spain | 4 | -0.74 (-0.94; -0.54)* | 1992 - 2006: 0.58 (0.51, 0.65)* 2006 - 2010: -3.22 (-3.88, -2.54)* 2010 - 2015: -5.83 (-6.28, -5.38)* 2015 - 2018: 4.05 (2.48, 5.64)* 2018 - 2021: 0.53 (-0.18, 1.25) |  | 3 | -0.72 (-1.00; -0.44)* | 1992 - 2006: 1.07 (0.95, 1.18)* 2006 - 2011: -3.65 (-4.35, -2.95)* 2011 - 2014: -8.19 (-10.46, -5.85)* 2014 - 2021: 1.20 (0.86, 1.54)* |
| Sweden | 3 | 0.40 (0.36; 0.44)* | 1992 - 1999: 0.89 (0.81, 0.96)* 1999 - 2012: 0.07 (0.04, 0.10)* 2012 - 2019: 1.14 (1.06, 1.22)* 2019 - 2021: -1.63 (-2.07, -1.19)* |  | 3 | 0.81 (0.70; 0.92)* | 1992 - 2000: 1.67 (1.50, 1.84)* 2000 - 2012: 0.05 (-0.05, 0.14) 2012 - 2019: 2.13 (1.92, 2.35)* 2019 - 2021: -2.55 (-3.72, -1.37)* |
| United Kingdom | 5 | -0.49 (-0.54; -0.44)* | 1992 - 1994: 2.82 (2.46, 3.17)* 1994 - 2006: 0.16 (0.14, 0.18)* 2006 - 2009: 2.20 (1.88, 2.51)* 2009 - 2015: -0.40 (-0.46, -0.33)* 2015 - 2019: -6.39 (-6.53, -6.26)* 2019 - 2021: 0.32 (0.00, 0.63)* |  | 5 | -0.43 (-0.53; -0.34)* | 1992 - 1994: 3.19 (2.48, 3.91)* 1994 - 2006: 0.26 (0.22, 0.31)* 2006 - 2009: 4.57 (3.93, 5.21)* 2009 - 2015: -0.18 (-0.31, -0.05)* 2015 - 2019: -8.47 (-8.74, -8.19)* 2019 - 2021: 0.56 (-0.08, 1.22) |
| CENTRAL/  EASTERN | 6 | 0.05 (-0.06; 0.16) | 1992 - 1998: 2.20 (2.08, 2.32)* 1998 - 2006: 0.56 (0.47, 0.64)* 2006 - 2009: 2.74 (2.12, 3.37)* 2009 - 2013: 0.89 (0.60, 1.18)* 2013 - 2016: -0.23 (-0.79, 0.33) 2016 - 2019: -3.77 (-4.31, -3.24)* 2019 - 2021: -7.46 (-8.00, -6.92)* |  | 4 | 0.18 (0.11; 0.25)* | 1992 - 1999: 1.97 (1.86, 2.07)* 1999 - 2005: 0.32 (0.16, 0.47)* 2005 - 2010: 2.18 (1.97, 2.40)* 2010 - 2019: 0.21 (0.14, 0.28)* 2019 - 2021: -10.86 (-11.44, -10.27)* |
| NORTHERN | 5 | -0.41 (-0.46; -0.37)* | 1992 - 1994: 2.21 (1.85, 2.58)* 1994 - 2006: 0.30 (0.28, 0.33)* 2006 - 2009: 1.19 (0.87, 1.51)* 2009 - 2015: -0.91 (-0.98, -0.84)* 2015 - 2019: -4.43 (-4.58, -4.28)* 2019 - 2021: 0.01 (-0.31, 0.33) |  | 5 | -0.36 (-0.42; -0.30)* | 1992 - 1994: 2.43 (1.99, 2.88)* 1994 - 2006: 0.59 (0.56, 0.62)* 2006 - 2009: 1.91 (1.52, 2.31)* 2009 - 2015: -0.78 (-0.86, -0.69)* 2015 - 2019: -5.72 (-5.90, -5.54)* 2019 - 2021: 0.14 (-0.26, 0.55) |
| SOUTHERN | 4 | -0.24 (-0.33; -0.15)* | 1992 - 2000: 0.66 (0.56, 0.75)* 2000 - 2006: 0.90 (0.72, 1.08)* 2006 - 2010: -0.69 (-1.08, -0.30)* 2010 - 2019: -1.61 (-1.70, -1.53)* 2019 - 2021: -0.07 (-0.85, 0.71) |  | 4 | -0.08 (-0.19; 0.02) | 1992 - 2000: 0.96 (0.85, 1.07)* 2000 - 2005: 1.37 (1.07, 1.68)* 2005 - 2009: -0.30 (-0.76, 0.16) 2009 - 2019: -1.61 (-1.69, -1.52)* 2019 - 2021: 0.28 (-0.65, 1.21) |
| WESTERN | 4 | 0.04 (-0.25; 0.32) | 1992 - 2001: 1.51 (1.24, 1.78)* 2001 - 2010: -2.21 (-2.50, -1.92)* 2010 - 2015: 8.01 (7.16, 8.88)* 2015 - 2019: -7.62 (-8.73, -6.49)* 2019 - 2021: 0.42 (-2.08, 2.97) |  | 4 | -0.02 (-0.22; 0.18) | 1992 - 2000: 0.83 (0.61, 1.05)* 2000 - 2010: -1.69 (-1.87, -1.52)* 2010 - 2015: 8.44 (7.83, 9.06)* 2015 - 2019: -7.75 (-8.55, -6.95)* 2019 - 2021: 0.82 (-0.99, 2.66) |
| UE28 | 5 | -0.16 (-0.23; -0.09)* | 1992 - 1999: 1.15 (1.06, 1.24)* 1999 - 2005: 0.15 (0.01, 0.28)* 2005 - 2010: -0.68 (-0.86, -0.49)* 2010 - 2015: 2.18 (2.00, 2.36)* 2015 - 2019: -4.63 (-4.89, -4.37)* 2019 - 2021: -0.93 (-1.47, -0.38)* |  | 4 | -0.08 (-0.15; 0.00) | 1992 - 1998: 1.23 (1.10, 1.35)* 1998 - 2011: 0.16 (0.12, 0.20)* 2011 - 2015: 2.41 (2.08, 2.74)* 2015 - 2019: -4.42 (-4.73, -4.11)* 2019 - 2021: -1.52 (-2.17, -0.87)* |

AAPC: Anual Average percentage change. JP: Joinpoint. APC: Annual Percentage Change and 95% confidence interval. * = p<0.05

Western countries: green, Southern countries: red, Northern countries: blue, Central and Eastern countries: yellow.
