# Supplementary material for: Incidence and mortality of nonmelanoma skin cancer in Europe: current trends and challenges
Source: Clin Transl Oncol. 2025 Jul 11;28(1):302–19. doi: 10.1007/s12094-025-03985-z (PMC12790528; doi:10.1007/s12094-025-03985-z)
Supplement: Supplementary file 7 — Supplementary file7 (DOCX 23 KB) [file 12094_2025_3985_MOESM7_ESM.docx]

**Supplementary table 3.** Results of Joinpoint Analysis for NMSC Incidence by Sex in ≥ 75 years old in European Countries (1992–2021).

| **Location** | **MEN** | | |  | **WOMEN** | | |
| --- | --- | --- | --- | --- | --- | --- | --- |
|  | **JP** | **AAPC 1992-2021** | **APC** |  | **JP** | **AAPC 1992-2021** | **APC** |
| Austria | 5 | 0.46 (0.32; 0.60)* | 1992 - 1999: -0.45 (-0.63, -0.27)* 1999 - 2005: 0.77 (0.50, 1.04)* 2005 - 2010: -1.80 (-2.15, -1.46)* 2010 - 2015: -0.31 (-0.63, 0.02) 2015 - 2018: 6.66 (5.72, 7.62)* 2018 - 2021: 1.05 (0.65, 1.46)* |  | 5 | 0.13 (0.05; 0.20)* | 1992 - 1994: -1.89 (-2.46, -1.31)* 1994 - 2006: 0.07 (0.03, 0.11)* 2006 - 2010: -3.22 (-3.48, -2.96)* 2010 - 2015: -0.89 (-1.06, -0.72)* 2015 - 2019: 5.93 (5.67, 6.19)* 2019 - 2021: 0.57 (0.11, 1.04)* |
| Belgium | 3 | 0.10 (0.07; 0.14)* | 1992 - 2006: 0.06 (0.04, 0.08)* 2006 - 2010: -1.59 (-1.78, -1.41)* 2010 - 2015: 1.84 (1.74, 1.95)* 2015 - 2021: -0.09 (-0.14, -0.04)* |  | 4 | 0.12 (0.08; 0.17)* | 1992 - 1994: -0.25 (-0.75, 0.24) 1994 - 2005: 0.25 (0.21, 0.28)* 2005 - 2010: -1.14 (-1.28, -1.01)* 2010 - 2015: 1.14 (1.01, 1.27)* 2015 - 2021: 0.24 (0.17, 0.31)* |
| Bulgaria | 3 | 0.58 (0.52; 0.64)* | 1992 - 1999: 4.72 (4.57, 4.86)* 1999 - 2016: -0.02 (-0.04, -0.00)* 2016 - 2019: 4.00 (3.55, 4.45)* 2019 - 2021: -12.59 (-12.98, -12.20)* |  | 1 | 1.09 (0.52; 1.65)* | 1992 - 1999: 4.12 (1.70, 6.61)* 1999 - 2021: 0.14 (-0.04, 0.32) |
| Croatia | 6 | 0.21 (0.20; 0.22)* | 1992 - 1995: -0.25 (-0.30, -0.19)* 1995 - 2000: 0.76 (0.73, 0.80)* 2000 - 2005: -0.03 (-0.06, -0.00)* 2005 - 2010: 0.27 (0.24, 0.30)* 2010 - 2015: 0.03 (0.01, 0.06)* 2015 - 2019: 0.46 (0.42, 0.49)* 2019 - 2021: -0.04 (-0.11, 0.02) |  | 6 | 0.08 (0.06; 0.10)* | 1992 - 1995: 0.08 (-0.02, 0.17) 1995 - 2000: -1.01 (-1.06, -0.95)* 2000 - 2005: 0.73 (0.68, 0.78)* 2005 - 2010: -0.42 (-0.47, -0.38)* 2010 - 2015: 0.23 (0.19, 0.27)* 2015 - 2018: 1.27 (1.15, 1.40)* 2018 - 2021: 0.20 (0.14, 0.26)* |
| Cyprus | 6 | 0.14 (0.11; 0.16)* | 1992 - 2000: -0.07 (-0.10, -0.05)* 2000 - 2005: 0.19 (0.14, 0.24)* 2005 - 2008: -0.17 (-0.34, -0.00)* 2008 - 2011: 0.05 (-0.09, 0.19) 2011 - 2014: 1.04 (0.94, 1.15)* 2014 - 2017: 0.25 (0.14, 0.36)* 2017 - 2021: 0.03 (-0.01, 0.07) |  | 3 | -0.14 (-0.18; -0.09)* | 1992 - 2006: -0.09 (-0.13, -0.06)* 2006 - 2010: 0.22 (-0.05, 0.50) 2010 - 2015: -1.09 (-1.21, -0.98)* 2015 - 2021: 0.33 (0.27, 0.38)* |
| Czechia | 3 | -1.10 (-1.33; -0.87)* | 1992 - 1999: 5.52 (5.13, 5.91)* 1999 - 2016: 1.52 (1.44, 1.59)* 2016 - 2019: -14.30 (-15.73, -12.86)* 2019 - 2021: -21.76 (-23.44, -20.03)* |  | 2 | -1.29 (-1.44; -1.13)* | 1992 - 2000: 4.15 (3.89, 4.42)* 2000 - 2019: 1.21 (1.15, 1.27)* 2019 - 2021: -37.19 (-38.48, -35.86)* |
| Denmark | 4 | -0.43 (-0.53; -0.32)* | 1992 - 2001: 1.46 (1.37, 1.55)* 2001 - 2008: 0.38 (0.23, 0.52)* 2008 - 2011: -1.21 (-2.01, -0.39)* 2011 - 2015: -5.49 (-5.89, -5.09)* 2015 - 2021: -0.32 (-0.45, -0.18)* |  | 6 | -0.54 (-0.66; -0.42)* | 1992 - 1999: 1.66 (1.56, 1.76)* 1999 - 2003: 1.05 (0.71, 1.39)* 2003 - 2006: 0.14 (-0.52, 0.81) 2006 - 2011: -3.06 (-3.27, -2.85)* 2011 - 2014: -4.37 (-5.07, -3.66)* 2014 - 2018: -1.26 (-1.63, -0.89)* 2018 - 2021: 0.69 (0.33, 1.05)* |
| Estonia | 4 | -0.26 (-0.31; -0.21)* | 1992 - 1999: 0.80 (0.74, 0.87)* 1999 - 2005: 1.73 (1.63, 1.84)* 2005 - 2011: -0.75 (-0.84, -0.66)* 2011 - 2014: -6.03 (-6.40, -5.66)* 2014 - 2021: -0.05 (-0.10, -0.00)* |  | 4 | -0.32 (-0.36; -0.28)* | 1992 - 2000: 0.95 (0.90, 0.99)* 2000 - 2005: 2.01 (1.89, 2.13)* 2005 - 2011: -0.75 (-0.83, -0.67)* 2011 - 2014: -6.81 (-7.13, -6.49)* 2014 - 2021: -0.16 (-0.20, -0.11)* |
| Finland | 4 | -0.04 (-0.05; -0.03)* | 1992 - 1995: 0.01 (-0.05, 0.06) 1995 - 2000: -0.19 (-0.22, -0.15)* 2000 - 2005: 0.25 (0.22, 0.28)* 2005 - 2015: -0.14 (-0.15, -0.13)* 2015 - 2021: -0.03 (-0.04, -0.02)* |  | 3 | 0.04 (-0.03; 0.10) | 1992 - 1994: -0.45 (-1.25, 0.35) 1994 - 2004: 0.18 (0.11, 0.24)* 2004 - 2018: -0.17 (-0.20, -0.13)* 2018 - 2021: 0.85 (0.54, 1.17)* |
| France | 5 | -0.55 (-0.77; -0.32)* | 1992 - 2000: 2.00 (1.85, 2.15)* 2000 - 2003: -1.75 (-2.90, -0.59)* 2003 - 2006: -3.14 (-4.27, -2.01)* 2006 - 2009: -5.03 (-6.14, -3.90)* 2009 - 2012: -0.65 (-1.82, 0.54) 2012 - 2021: 0.06 (-0.04, 0.16) |  | 5 | -0.97 (-1.05; -0.88)* | 1992 - 1997: 0.12 (-0.04, 0.28) 1997 - 2001: -1.31 (-1.64, -0.97)* 2001 - 2005: -4.19 (-4.52, -3.86)* 2005 - 2009: -2.06 (-2.40, -1.72)* 2009 - 2015: -0.32 (-0.47, -0.16)* 2015 - 2021: 0.63 (0.52, 0.74)* |
| Germany | 3 | 0.35 (-0.14; 0.84) | 1992 - 2010: 0.63 (0.40, 0.86)* 2010 - 2015: 14.56 (12.90, 16.24)* 2015 - 2019: -15.92 (-17.65, -14.16)* 2019 - 2021: 0.12 (-4.54, 5.01) |  | 4 | 0.58 (0.25; 0.90)* | 1992 - 2004: 1.08 (0.86, 1.30)* 2004 - 2010: -0.56 (-1.28, 0.16) 2010 - 2015: 12.89 (11.94, 13.84)* 2015 - 2019: -12.95 (-14.03, -11.86)* 2019 - 2021: 1.00 (-1.86, 3.94) |
| Greece | 6 | 0.03 (0.03; 0.04)* | 1992 - 1995: 0.11 (0.09, 0.12)* 1995 - 2000: 0.03 (0.02, 0.04)* 2000 - 2005: 0.14 (0.13, 0.15)* 2005 - 2010: -0.11 (-0.12, -0.11)* 2010 - 2015: 0.18 (0.17, 0.19)* 2015 - 2019: -0.15 (-0.17, -0.14)* 2019 - 2021: 0.02 (-0.00, 0.04) |  | 4 | 0.07 (0.05; 0.09)* | 1992 - 1997: -0.03 (-0.09, 0.03) 1997 - 2005: 0.28 (0.25, 0.32)* 2005 - 2010: -0.21 (-0.29, -0.14)* 2010 - 2019: -0.05 (-0.07, -0.03)* 2019 - 2021: 0.74 (0.56, 0.91)* |
| Hungary | 3 | 0.04 (0.03; 0.06)* | 1992 - 1995: 1.24 (1.15, 1.34)* 1995 - 2000: -0.74 (-0.80, -0.69)* 2000 - 2004: 0.20 (0.11, 0.28)* 2004 - 2021: 0.03 (0.02, 0.03)* |  | 2 | -0.01 (-0.04; 0.03) | 1992 - 1996: 0.39 (0.29, 0.50)* 1996 - 1999: -1.01 (-1.32, -0.69)* 1999 - 2021: 0.06 (0.05, 0.06)* |
| Ireland | 4 | -1.34 (-1.38; -1.29)* | 1992 - 2003: 1.09 (1.07, 1.12)* 2003 - 2006: -0.29 (-0.59, 0.02) 2006 - 2010: -6.58 (-6.73, -6.44)* 2010 - 2014: -5.35 (-5.50, -5.19)* 2014 - 2021: -0.12 (-0.16, -0.08)* |  | 5 | -1.67 (-1.87; -1.47)* | 1992 - 2000: 1.10 (0.95, 1.26)* 2000 - 2003: 3.82 (2.55, 5.10)* 2003 - 2006: 0.24 (-0.90, 1.40) 2006 - 2010: -12.03 (-12.59, -11.47)* 2010 - 2014: -4.66 (-5.35, -3.97)* 2014 - 2021: 0.11 (-0.08, 0.30) |
| Italy | 3 | 0.15 (0.09; 0.21)* | 1992 - 2008: 1.04 (1.01, 1.07)* 2008 - 2015: 0.13 (0.03, 0.23)* 2015 - 2019: -3.35 (-3.62, -3.08)* 2019 - 2021: 0.25 (-0.31, 0.81) |  | 3 | 0.43 (0.40; 0.45)* | 1992 - 2009: 0.91 (0.90, 0.93)* 2009 - 2015: 0.07 (0.01, 0.13)* 2015 - 2019: -1.42 (-1.55, -1.30)* 2019 - 2021: 1.11 (0.85, 1.37)* |
| Latvia | 3 | -0.02 (-0.04; -0.00)* | 1992 - 2000: 0.03 (-0.00, 0.06) 2000 - 2010: 0.43 (0.40, 0.45)* 2010 - 2015: -1.27 (-1.36, -1.19)* 2015 - 2021: 0.21 (0.17, 0.25)* |  | 4 | 0.19 (0.17; 0.22)* | 1992 - 2001: -0.03 (-0.05, -0.01)* 2001 - 2005: 1.06 (0.95, 1.17)* 2005 - 2010: 0.78 (0.72, 0.85)* 2010 - 2015: -0.39 (-0.46, -0.33)* 2015 - 2021: -0.04 (-0.07, -0.00)* |
| Lithuania | 4 | 0.05 (0.02; 0.09)* | 1992 - 2000: -0.01 (-0.05, 0.03) 2000 - 2005: 1.86 (1.74, 1.98)* 2005 - 2010: 0.98 (0.87, 1.08)* 2010 - 2015: -2.46 (-2.56, -2.36)* 2015 - 2021: 0.01 (-0.05, 0.06) |  | 4 | -0.24 (-0.33; -0.15)* | 1992 - 2000: -0.02 (-0.10, 0.06) 2000 - 2008: 1.66 (1.57, 1.75)* 2008 - 2011: -0.17 (-0.77, 0.43) 2011 - 2014: -5.94 (-6.51, -5.37)* 2014 - 2021: -0.15 (-0.23, -0.07)* |
| Luxembourg | 5 | 0.05 (0.05; 0.05)* | 1992 - 1995: 0.04 (0.02, 0.06)* 1995 - 2000: -0.12 (-0.13, -0.11)* 2000 - 2005: 0.38 (0.36, 0.39)* 2005 - 2010: -0.16 (-0.17, -0.15)* 2010 - 2015: 0.17 (0.16, 0.18)* 2015 - 2021: 0.00 (-0.00, 0.01) |  | 3 | 0.06 (0.01; 0.11)* | 1992 - 1996: -0.14 (-0.40, 0.12) 1996 - 2006: 0.18 (0.12, 0.25)* 2006 - 2018: -0.17 (-0.22, -0.13)* 2018 - 2021: 0.84 (0.53, 1.15)* |
| Malta | 3 | -1.03 (-1.21; -0.84)* | 1992 - 2011: 0.38 (0.32, 0.44)* 2011 - 2015: -2.67 (-3.45, -1.89)* 2015 - 2019: -6.70 (-7.47, -5.91)* 2019 - 2021: 0.74 (-0.85, 2.35) |  | 4 | -0.37 (-0.46; -0.27)* | 1992 - 2002: 0.14 (0.05, 0.23)* 2002 - 2010: 1.07 (0.94, 1.20)* 2010 - 2015: -1.18 (-1.44, -0.92)* 2015 - 2019: -4.01 (-4.41, -3.61)* 2019 - 2021: 0.85 (0.05, 1.66)* |
| Netherlands | 3 | 0.35 (0.23; 0.47)* | 1992 - 1999: 2.84 (2.58, 3.11)* 1999 - 2007: 0.28 (0.06, 0.50)* 2007 - 2019: -0.39 (-0.48, -0.29)* 2019 - 2021: -3.48 (-4.68, -2.27)* |  | 4 | 0.04 (-0.04; 0.13) | 1992 - 2000: 2.28 (2.18, 2.38)* 2000 - 2010: -0.62 (-0.69, -0.55)* 2010 - 2014: -1.21 (-1.58, -0.83)* 2014 - 2019: -0.07 (-0.30, 0.17) 2019 - 2021: -2.62 (-3.34, -1.90)* |
| Poland | 4 | 1.04 (1.02; 1.06)* | 1992 - 2003: -0.12 (-0.13, -0.11)* 2003 - 2006: 1.21 (1.08, 1.35)* 2006 - 2009: 8.88 (8.75, 9.01)* 2009 - 2012: 0.78 (0.68, 0.88)* 2012 - 2021: -0.00 (-0.01, 0.00) |  | 4 | 1.00 (0.96; 1.04)* | 1992 - 2003: -0.09 (-0.11, -0.07)* 2003 - 2006: 0.94 (0.68, 1.21)* 2006 - 2009: 8.21 (7.96, 8.46)* 2009 - 2012: 0.84 (0.63, 1.04)* 2012 - 2021: 0.10 (0.08, 0.11)* |
| Portugal | 6 | 2.07 (1.98; 2.16)* | 1992 - 1996: 0.56 (0.28, 0.85)* 1996 - 2001: 2.14 (1.90, 2.39)* 2001 - 2004: 4.41 (3.76, 5.07)* 2004 - 2010: 1.79 (1.67, 1.90)* 2010 - 2014: 5.10 (4.88, 5.32)* 2014 - 2017: 1.03 (0.66, 1.41)* 2017 - 2021: 0.01 (-0.10, 0.12) |  | 4 | 0.70 (0.64; 0.76)* | 1992 - 1995: -0.17 (-0.51, 0.18) 1995 - 2004: 1.21 (1.15, 1.28)* 2004 - 2011: 0.32 (0.24, 0.40)* 2011 - 2014: 2.23 (1.79, 2.68)* 2014 - 2021: 0.15 (0.09, 0.20)* |
| Romania | 3 | 0.90 (0.86; 0.94)* | 1992 - 1996: 3.92 (3.79, 4.06)* 1996 - 1999: 3.27 (2.89, 3.65)* 1999 - 2012: 0.12 (0.10, 0.14)* 2012 - 2021: -0.07 (-0.10, -0.05)* |  | 3 | 0.88 (0.83; 0.92)* | 1992 - 1999: 3.38 (3.31, 3.44)* 1999 - 2002: 0.26 (-0.12, 0.64) 2002 - 2014: 0.00 (-0.02, 0.02) 2014 - 2021: 0.18 (0.14, 0.22)* |
| Slovakia | 3 | -0.25 (-0.31; -0.18)* | 1992 - 2005: 0.64 (0.60, 0.69)* 2005 - 2010: 3.68 (3.42, 3.93)* 2010 - 2015: -6.70 (-6.93, -6.47)* 2015 - 2021: 0.19 (0.05, 0.33)* |  | 3 | -0.38 (-0.45; -0.32)* | 1992 - 2005: 0.13 (0.08, 0.18)* 2005 - 2010: 3.65 (3.40, 3.90)* 2010 - 2015: -6.38 (-6.61, -6.16)* 2015 - 2021: 0.38 (0.24, 0.51)* |
| Slovenia | 2 | 1.46 (1.38; 1.54)* | 1992 - 1999: 6.95 (6.73, 7.17)* 1999 - 2019: 0.92 (0.89, 0.95)* 2019 - 2021: -10.98 (-11.76, -10.20)* |  | 2 | 0.68 (0.54; 0.81)* | 1992 - 2000: 4.94 (4.66, 5.22)* 2000 - 2019: 0.75 (0.69, 0.81)* 2019 - 2021: -15.27 (-16.66, -13.85)* |
| Spain | 5 | -0.22 (-0.32; -0.13)* | 1992 - 1999: 0.40 (0.28, 0.52)* 1999 - 2005: 1.47 (1.30, 1.64)* 2005 - 2009: -1.79 (-2.13, -1.45)* 2009 - 2015: -3.57 (-3.72, -3.42)* 2015 - 2018: 3.24 (2.54, 3.94)* 2018 - 2021: 0.47 (0.15, 0.79)* |  | 3 | -0.45 (-0.55; -0.36)* | 1992 - 1998: 0.31 (0.03, 0.58)* 1998 - 2006: 0.90 (0.72, 1.09)* 2006 - 2015: -3.41 (-3.54, -3.27)* 2015 - 2021: 1.51 (1.28, 1.73)* |
| Sweden | 3 | 0.25 (0.06; 0.45)* | 1992 - 1997: 4.69 (4.02, 5.37)* 1997 - 2012: 0.08 (-0.03, 0.19) 2012 - 2019: 1.34 (0.99, 1.70)* 2019 - 2021: -12.27 (-14.06, -10.45)* |  | 3 | 0.76 (0.54; 0.98)* | 1992 - 1997: 5.45 (4.76, 6.15)* 1997 - 2013: 0.16 (0.05, 0.27)* 2013 - 2019: 2.52 (1.97, 3.07)* 2019 - 2021: -10.43 (-12.53, -8.29)* |
| United Kingdom | 5 | -0.39 (-0.45; -0.34)* | 1992 - 1994: 2.70 (2.21, 3.19)* 1994 - 2006: 0.12 (0.10, 0.15)* 2006 - 2009: 2.02 (1.65, 2.39)* 2009 - 2015: -0.31 (-0.39, -0.24)* 2015 - 2019: -5.58 (-5.74, -5.42)* 2019 - 2021: 0.30 (-0.04, 0.64) |  | 5 | -0.24 (-0.33; -0.15)* | 1992 - 1994: 2.88 (2.21, 3.57)* 1994 - 2006: 0.24 (0.19, 0.28)* 2006 - 2009: 3.38 (2.78, 3.99)* 2009 - 2015: -0.16 (-0.28, -0.03)* 2015 - 2019: -6.37 (-6.65, -6.09)* 2019 - 2021: 0.96 (0.35, 1.58)* |
| CENTRAL/  EASTERN | 5 | 0.21 (0.12; 0.30)* | 1992 - 1999: 3.33 (3.21, 3.45)* 1999 - 2005: 0.13 (-0.03, 0.30) 2005 - 2010: 1.71 (1.49, 1.92)* 2010 - 2016: 0.16 (0.03, 0.29)* 2016 - 2019: -3.56 (-4.09, -3.01)* 2019 - 2021: -7.77 (-8.31, -7.22)* |  | 2 | 0.21 (-0.06; 0.48) | 1992 - 1998: 2.65 (1.84, 3.46)* 1998 - 2019: 0.64 (0.54, 0.73)* 2019 - 2021: -10.85 (-13.74, -7.87)* |
| NORTHERN | 4 | -0.31 (-0.52; -0.10)* | 1992 - 1995: 2.68 (1.61, 3.77)* 1995 - 2010: 0.33 (0.24, 0.41)* 2010 - 2015: -0.60 (-1.08, -0.12)* 2015 - 2018: -3.87 (-5.31, -2.41)* 2018 - 2021: -2.30 (-3.04, -1.55)* |  | 4 | -0.19 (-0.51; 0.12) | 1992 - 1995: 2.58 (1.19, 3.99)* 1995 - 2010: 0.49 (0.37, 0.61)* 2010 - 2015: -0.48 (-1.22, 0.26) 2015 - 2018: -4.17 (-6.43, -1.86)* 2018 - 2021: -1.79 (-2.99, -0.58)* |
| SOUTHERN | 4 | 0.07 (-0.03; 0.18) | 1992 - 2000: 0.79 (0.67, 0.90)* 2000 - 2005: 1.27 (0.98, 1.56)* 2005 - 2008: -0.12 (-0.93, 0.70) 2008 - 2019: -0.87 (-0.93, -0.81)* 2019 - 2021: -0.24 (-0.94, 0.47) |  | 5 | 0.07 (-0.02; 0.16) | 1992 - 2000: 0.72 (0.63, 0.81)* 2000 - 2005: 1.00 (0.78, 1.21)* 2005 - 2008: -0.44 (-1.07, 0.19) 2008 - 2014: -1.13 (-1.27, -1.00)* 2014 - 2018: -0.37 (-0.66, -0.07)* 2018 - 2021: 0.31 (0.02, 0.61)* |
| WESTERN | 4 | -0.08 (-0.28; 0.12) | 1992 - 2002: 1.46 (1.27, 1.66)* 2002 - 2010: -2.24 (-2.51, -1.96)* 2010 - 2015: 7.47 (6.87, 8.08)* 2015 - 2019: -8.45 (-9.19, -7.71)* 2019 - 2021: 0.27 (-1.42, 1.98) |  | 4 | -0.16 (-0.26; -0.07)* | 1992 - 2000: 0.44 (0.33, 0.56)* 2000 - 2010: -1.31 (-1.40, -1.22)* 2010 - 2015: 5.98 (5.69, 6.27)* 2015 - 2019: -6.33 (-6.71, -5.94)* 2019 - 2021: 1.02 (0.14, 1.90)* |
| UE28 | 4 | -0.07 (-0.21; 0.07) | 1992 - 2000: 1.49 (1.31, 1.68)* 2000 - 2011: -0.27 (-0.37, -0.16)* 2011 - 2015: 2.55 (1.96, 3.15)* 2015 - 2019: -4.63 (-5.16, -4.10)* 2019 - 2021: -1.00 (-2.12, 0.13) |  | 5 | -0.10 (-0.15; -0.05)* | 1992 - 1994: 1.89 (1.47, 2.31)* 1994 - 2000: 0.61 (0.52, 0.69)* 2000 - 2011: -0.19 (-0.22, -0.17)* 2011 - 2015: 1.79 (1.63, 1.95)* 2015 - 2018: -3.35 (-3.64, -3.05)* 2018 - 2021: -1.63 (-1.79, -1.47)* |

AAPC: Anual Average percentage change. JP: Joinpoint. APC: Annual Percentage Change and 95% confidence interval. * = p<0.05

Western countries: green, Southern countries: red, Northern countries: blue, Central and Eastern countries: yellow.
