# Supplementary material for: Incidence and mortality of nonmelanoma skin cancer in Europe: current trends and challenges
Source: Clin Transl Oncol. 2025 Jul 11;28(1):302–19. doi: 10.1007/s12094-025-03985-z (PMC12790528; doi:10.1007/s12094-025-03985-z)
Supplement: Supplementary file 8 — Supplementary file8 (DOCX 22 KB) [file 12094_2025_3985_MOESM8_ESM.docx]

**Supplementary table 4.** Results of Joinpoint Analysis for SCC Mortality by Sex in <45 years old in European Countries (1992–2021).

| **Location** | **MEN** | | |  | **WOMEN** | | |
| --- | --- | --- | --- | --- | --- | --- | --- |
|  | **JP** | **AAPC 1992-2021** | **APC** |  | **JP** | **AAPC 1992-2021** | **APC** |
| Austria | 4 | -1.34 (-2.21; -0.45)* | 1992 - 1999: -4.68 (-5.48, -3.89)* 1999 - 2002: 1.95 (-4.31, 8.62) 2002 - 2015: -1.08 (-1.47, -0.69)* 2015 - 2019: 5.38 (1.91, 8.96)* 2019 - 2021: -8.66 (-14.64, -2.27)* |  | 4 | -1.69 (-2.29; -1.09)* | 1992 - 1997: -5.30 (-6.39, -4.19)* 1997 - 2001: -0.17 (-2.86, 2.60) 2001 - 2008: -3.30 (-4.22, -2.38)* 2008 - 2019: 1.57 (1.10, 2.04)* 2019 - 2021: -7.32 (-12.75, -1.56)* |
| Belgium | 0 | -0.42 (-0.68; -0.16)* | 1992 - 2021: -0.42 (-0.68, -0.16)* |  | 1 | -1.31 (-1.65; -0.96)* | 1992 - 2003: -2.09 (-2.80, -1.38)* 2003 - 2021: -0.82 (-1.21, -0.44)* |
| Bulgaria | 0 | -1.28 (-1.69; -0.87)* | 1992 - 2021: -1.28 (-1.69, -0.87)* |  | 3 | -3.11 (-4.91; -1.27)* | 1992 - 2001: -0.67 (-1.82, 0.49) 2001 - 2012: -3.92 (-5.05, -2.77)* 2012 - 2015: 9.09 (-8.41, 29.94) 2015 - 2021: -10.66 (-13.44, -7.79)* |
| Croatia | 2 | -3.30 (-4.90; -1.67)* | 1992 - 2013: -2.98 (-3.30, -2.66)* 2013 - 2016: 4.51 (-10.55, 22.11) 2016 - 2021: -8.98 (-12.53, -5.27)* |  | 3 | -4.55 (-6.49; -2.57)* | 1992 - 2003: -3.02 (-3.82, -2.22)* 2003 - 2012: -5.50 (-7.16, -3.82)* 2012 - 2015: 6.45 (-12.02, 28.81) 2015 - 2021: -10.87 (-13.95, -7.67)* |
| Cyprus | 2 | -1.22 (-2.08; -0.36)* | 1992 - 2004: -0.62 (-1.39, 0.15) 2004 - 2008: 2.48 (-3.43, 8.75) 2008 - 2021: -2.88 (-3.47, -2.28)* |  | 1 | -2.07 (-2.56; -1.58)* | 1992 - 1999: 0.69 (-1.21, 2.64) 1999 - 2021: -2.94 (-3.26, -2.62)* |
| Czechia | 3 | -4.47 (-6.28; -2.62)* | 1992 - 2013: -3.37 (-3.62, -3.13)* 2013 - 2016: 7.40 (-3.64, 19.71) 2016 - 2019: -21.08 (-30.31, -10.62)* 2019 - 2021: -5.36 (-19.44, 11.19) |  | 3 | -5.58 (-7.68; -3.42)* | 1992 - 2007: -3.52 (-4.11, -2.93)* 2007 - 2012: -8.16 (-13.25, -2.77)* 2012 - 2015: 9.39 (-10.06, 33.05) 2015 - 2021: -14.93 (-18.12, -11.60)* |
| Denmark | 1 | -1.15 (-1.99; -0.30)* | 1992 - 1994: 4.78 (-7.59, 18.80) 1994 - 2021: -1.57 (-1.81, -1.34)* |  | 2 | -2.02 (-2.41; -1.63)* | 1992 - 1995: -0.24 (-3.27, 2.89) 1995 - 2011: -3.01 (-3.29, -2.73)* 2011 - 2021: -0.95 (-1.59, -0.31)* |
| Estonia | 3 | -3.52 (-4.17; -2.87)* | 1992 - 1999: 0.91 (-0.12, 1.96) 1999 - 2004: -6.37 (-8.85, -3.83)* 2004 - 2013: -2.45 (-3.49, -1.40)* 2013 - 2021: -6.66 (-7.90, -5.41)* |  | 1 | -3.58 (-4.10; -3.05)* | 1992 - 2000: 0.27 (-1.27, 1.84) 2000 - 2021: -5.00 (-5.50, -4.50)* |
| Finland | 4 | -1.34 (-2.04; -0.64)* | 1992 - 1998: -3.46 (-4.40, -2.51)* 1998 - 2009: 0.60 (0.10, 1.10)* 2009 - 2014: -3.17 (-5.21, -1.10)* 2014 - 2019: 0.65 (-1.52, 2.88) 2019 - 2021: -5.73 (-12.08, 1.07) |  | 1 | -1.44 (-1.79; -1.08)* | 1992 - 1996: -3.51 (-5.90, -1.06)* 1996 - 2021: -1.10 (-1.28, -0.92)* |
| France | 3 | -1.14 (-2.34; 0.08) | 1992 - 1998: -3.11 (-4.90, -1.29)* 1998 - 2001: 10.98 (-0.68, 24.01) 2001 - 2013: -3.20 (-3.92, -2.48)* 2013 - 2021: -0.79 (-2.17, 0.60) |  | 4 | -1.57 (-2.26; -0.87)* | 1992 - 1994: -4.21 (-9.16, 1.00) 1994 - 2002: -0.58 (-1.32, 0.16) 2002 - 2006: -3.56 (-6.34, -0.69)* 2006 - 2019: -0.61 (-0.98, -0.24)* 2019 - 2021: -4.95 (-11.16, 1.70) |
| Germany | 1 | -1.45 (-1.97; -0.92)* | 1992 - 1999: -4.07 (-5.93, -2.18)* 1999 - 2021: -0.60 (-0.99, -0.21)* |  | 1 | -1.24 (-1.50; -0.98)* | 1992 - 2006: -2.73 (-3.10, -2.36)* 2006 - 2021: 0.18 (-0.22, 0.58) |
| Greece | 0 | 0.15 (-0.10; 0.39) | 1992 - 2021: 0.15 (-0.10, 0.39) |  | 2 | -0.55 (-0.87; -0.23)* | 1992 - 2006: -1.24 (-1.52, -0.97)* 2006 - 2016: 1.25 (0.70, 1.81)* 2016 - 2021: -2.17 (-3.56, -0.76)* |
| Hungary | 3 | -5.61 (-7.25; -3.94)* | 1992 - 2006: -3.51 (-4.01, -3.02)* 2006 - 2012: -8.60 (-11.46, -5.65)* 2012 - 2015: 9.22 (-6.58, 27.70) 2015 - 2021: -13.91 (-16.48, -11.27)* |  | 3 | -7.01 (-9.83; -4.11)* | 1992 - 2009: -4.98 (-5.48, -4.47)* 2009 - 2012: -12.86 (-29.10, 7.09) 2012 - 2015: 11.61 (-10.55, 39.26) 2015 - 2021: -17.54 (-21.20, -13.70)* |
| Ireland | 5 | -1.31 (-1.96; -0.65)* | 1992 - 1998: -0.09 (-0.90, 0.73) 1998 - 2001: 4.94 (0.34, 9.75)* 2001 - 2005: -3.41 (-5.42, -1.35)* 2005 - 2009: 2.91 (0.86, 5.01)* 2009 - 2019: -3.19 (-3.55, -2.82)* 2019 - 2021: -8.26 (-12.65, -3.66)* |  | 5 | -1.82 (-2.58; -1.06)* | 1992 - 1998: -1.42 (-2.25, -0.59)* 1998 - 2001: 2.56 (-2.31, 7.67) 2001 - 2004: -6.20 (-10.57, -1.61)* 2004 - 2010: 0.32 (-0.73, 1.39) 2010 - 2019: -2.20 (-2.71, -1.70)* 2019 - 2021: -7.24 (-12.01, -2.20)* |
| Italy | 3 | -1.02 (-1.96; -0.07)* | 1992 - 1995: 7.38 (0.90, 14.27)* 1995 - 2006: -4.37 (-5.30, -3.43)* 2006 - 2016: 1.94 (0.71, 3.18)* 2016 - 2021: -4.12 (-7.18, -0.96)* |  | 3 | -1.63 (-2.52; -0.74)* | 1992 - 2000: -0.92 (-2.07, 0.25) 2000 - 2009: -5.37 (-6.58, -4.14)* 2009 - 2014: 5.35 (1.19, 9.68)* 2014 - 2021: -2.38 (-4.12, -0.60)* |
| Latvia | 4 | -1.40 (-2.74; -0.04)* | 1992 - 1996: 0.64 (-2.06, 3.41) 1996 - 1999: 5.30 (-3.13, 14.46) 1999 - 2003: -6.11 (-10.09, -1.95)* 2003 - 2006: 3.57 (-5.50, 13.50) 2006 - 2021: -2.90 (-3.35, -2.45)* |  | 3 | -2.41 (-3.82; -0.98)* | 1992 - 2000: 2.18 (1.13, 3.24)* 2000 - 2003: -6.76 (-15.57, 2.97) 2003 - 2006: 1.87 (-8.35, 13.24) 2006 - 2021: -4.72 (-5.28, -4.16)* |
| Lithuania | 3 | -1.21 (-2.04; -0.38)* | 1992 - 1999: 0.48 (-0.48, 1.46) 1999 - 2002: -5.20 (-11.85, 1.95) 2002 - 2007: 6.18 (3.80, 8.61)* 2007 - 2021: -3.70 (-4.09, -3.30)* |  | 3 | -2.40 (-3.80; -0.97)* | 1992 - 2000: 0.85 (-0.40, 2.11) 2000 - 2003: -6.44 (-16.71, 5.10) 2003 - 2007: 5.87 (-0.31, 12.43) 2007 - 2021: -5.55 (-6.26, -4.83)* |
| Luxembourg | 4 | -3.50 (-4.43; -2.57)* | 1992 - 1999: -5.01 (-6.11, -3.89)* 1999 - 2003: 1.80 (-2.68, 6.48) 2003 - 2012: -4.83 (-5.83, -3.83)* 2012 - 2018: 0.26 (-1.98, 2.55) 2018 - 2021: -9.99 (-14.53, -5.21)* |  | 0 | -2.80 (-3.12; -2.49)* | 1992 - 2021: -2.80 (-3.12, -2.49)* |
| Malta | 5 | 0.63 (-0.27; 1.53) | 1992 - 1995: 4.07 (0.16, 8.13)* 1995 - 1999: -4.50 (-8.13, -0.72)* 1999 - 2009: 3.66 (2.95, 4.36)* 2009 - 2014: -2.02 (-4.15, 0.17) 2014 - 2018: 3.64 (0.24, 7.16)* 2018 - 2021: -5.03 (-8.18, -1.78)* |  | 4 | -0.85 (-1.98; 0.29) | 1992 - 1998: -6.22 (-7.75, -4.66)* 1998 - 2002: 3.83 (-1.57, 9.52) 2002 - 2007: -2.42 (-5.66, 0.93) 2007 - 2019: 2.44 (1.74, 3.14)* 2019 - 2021: -8.63 (-17.20, 0.84) |
| Netherlands | 4 | -0.42 (-1.26; 0.43) | 1992 - 1998: -1.29 (-2.41, -0.14)* 1998 - 2001: 3.48 (-3.25, 10.68) 2001 - 2012: -1.57 (-2.09, -1.04)* 2012 - 2018: 3.07 (1.42, 4.75)* 2018 - 2021: -5.01 (-8.50, -1.40)* |  | 2 | -1.13 (-1.82; -0.45)* | 1992 - 2004: -1.39 (-1.75, -1.02)* 2004 - 2007: -5.03 (-11.22, 1.59) 2007 - 2021: -0.06 (-0.41, 0.29) |
| Poland | 2 | -8.34 (-10.35; -6.29)* | 1992 - 2006: -2.16 (-2.88, -1.44)* 2006 - 2017: -17.12 (-19.02, -15.17)* 2017 - 2021: -3.79 (-17.65, 12.40) |  | 2 | -9.45 (-11.81; -7.02)* | 1992 - 2006: -4.11 (-4.74, -3.47)* 2006 - 2018: -18.55 (-20.27, -16.80)* 2018 - 2021: 5.91 (-17.91, 36.65) |
| Portugal | 6 | -2.31 (-3.88; -0.72)* | 1992 - 1998: -1.76 (-3.17, -0.33)* 1998 - 2001: 3.79 (-4.47, 12.77) 2001 - 2006: -4.76 (-7.27, -2.18)* 2006 - 2009: 4.06 (-4.48, 13.37) 2009 - 2015: -5.85 (-7.78, -3.88)* 2015 - 2018: 3.03 (-6.72, 13.79) 2018 - 2021: -9.12 (-14.04, -3.92)* |  | 1 | -1.69 (-2.02; -1.36)* | 1992 - 2006: -2.67 (-3.15, -2.19)* 2006 - 2021: -0.77 (-1.27, -0.27)* |
| Romania | 2 | -2.68 (-4.31; -1.02)* | 1992 - 2012: -2.62 (-2.99, -2.25)* 2012 - 2015: 5.74 (-9.94, 24.14) 2015 - 2021: -6.82 (-9.50, -4.07)* |  | 3 | -3.30 (-5.40; -1.14)* | 1992 - 1995: 1.32 (-5.31, 8.43) 1995 - 2012: -4.41 (-5.00, -3.81)* 2012 - 2015: 11.12 (-9.20, 35.98) 2015 - 2021: -8.93 (-12.15, -5.60)* |
| Slovakia | 5 | -2.26 (-2.75; -1.77)* | 1992 - 1995: -3.76 (-5.30, -2.18)* 1995 - 1998: -0.10 (-3.40, 3.31) 1998 - 2008: -1.76 (-2.08, -1.44)* 2008 - 2013: -4.19 (-5.37, -2.99)* 2013 - 2018: -0.28 (-1.53, 0.99) 2018 - 2021: -4.56 (-6.52, -2.56)* |  | 2 | -2.56 (-3.18; -1.95)* | 1992 - 2014: -2.77 (-2.91, -2.62)* 2014 - 2019: -0.24 (-2.48, 2.05) 2019 - 2021: -6.01 (-12.97, 1.51) |
| Slovenia | 1 | -5.62 (-6.84; -4.37)* | 1992 - 2016: -3.81 (-4.24, -3.38)* 2016 - 2021: -13.80 (-20.18, -6.91)* |  | 3 | -7.92 (-9.46; -6.36)* | 1992 - 2004: -4.57 (-5.35, -3.79)* 2004 - 2009: -9.77 (-14.85, -4.38)* 2009 - 2015: 0.84 (-3.85, 5.77) 2015 - 2021: -20.39 (-24.39, -16.17)* |
| Spain | 5 | -2.85 (-3.95; -1.74)* | 1992 - 1995: 1.26 (-2.58, 5.26) 1995 - 1999: -7.69 (-11.31, -3.91)* 1999 - 2003: 0.33 (-3.67, 4.50) 2003 - 2014: -4.14 (-4.77, -3.51)* 2014 - 2018: 0.86 (-3.89, 5.85) 2018 - 2021: -4.50 (-9.36, 0.62) |  | 1 | -1.78 (-2.08; -1.47)* | 1992 - 2006: -2.67 (-3.11, -2.22)* 2006 - 2021: -0.94 (-1.39, -0.49)* |
| Sweden | 7 | -1.53 (-3.91; 0.91) | 1992 - 1994: 3.18 (-7.83, 15.50) 1994 - 1999: -15.12 (-18.77, -11.30)* 1999 - 2002: 16.01 (0.50, 33.92)* 2002 - 2005: -5.76 (-17.93, 8.21) 2005 - 2008: 9.41 (-4.09, 24.80) 2008 - 2013: -5.91 (-9.85, -1.80)* 2013 - 2018: 10.39 (6.08, 14.87)* 2018 - 2021: -13.00 (-18.32, -7.32)* |  | 0 | 0.36 (-0.07; 0.79) | 1992 - 2021: 0.36 (-0.07, 0.79) |
| United Kingdom | 3 | 0.30 (-1.01; 1.63) | 1992 - 1999: -3.19 (-4.91, -1.43)* 1999 - 2013: 0.78 (0.06, 1.49)* 2013 - 2016: 20.06 (6.68, 35.11)* 2016 - 2021: -6.63 (-8.87, -4.34)* |  | 2 | 0.40 (-0.58; 1.38) | 1992 - 2012: -0.32 (-0.70, 0.05) 2012 - 2016: 11.45 (4.51, 18.85)* 2016 - 2021: -4.94 (-7.44, -2.37)* |
| CENTRAL/  EASTERN | 1 | -4.26 (-4.79; -3.73)* | 1992 - 2005: -2.51 (-3.29, -1.73)* 2005 - 2021: -5.66 (-6.44, -4.88)* |  | 3 | -5.69 (-7.59; -3.76)* | 1992 - 2006: -3.73 (-4.26, -3.20)* 2006 - 2012: -9.66 (-12.81, -6.38)* 2012 - 2015: 4.94 (-12.58, 25.96) 2015 - 2021: -11.04 (-14.07, -7.90)* |
| NORTHERN | 3 | -0.41 (-1.19; 0.39) | 1992 - 1999: -2.71 (-4.00, -1.41)* 1999 - 2013: 0.35 (-0.18, 0.89) 2013 - 2017: 9.88 (5.04, 14.96)* 2017 - 2021: -8.41 (-11.05, -5.68)* |  | 2 | -0.69 (-1.55; 0.17) | 1992 - 2013: -0.91 (-1.12, -0.70)* 2013 - 2016: 7.43 (-1.05, 16.63) 2016 - 2021: -4.39 (-6.12, -2.62)* |
| SOUTHERN | 5 | -1.85 (-2.60; -1.10)* | 1992 - 1995: 3.42 (1.04, 5.86)* 1995 - 1999: -5.59 (-7.78, -3.35)* 1999 - 2002: 0.72 (-3.97, 5.64) 2002 - 2006: -4.09 (-6.39, -1.73)* 2006 - 2019: -1.01 (-1.31, -0.71)* 2019 - 2021: -6.54 (-12.08, -0.65)* |  | 3 | -1.84 (-2.46; -1.22)* | 1992 - 2002: -1.95 (-2.44, -1.46)* 2002 - 2006: -5.46 (-8.65, -2.17)* 2006 - 2018: 0.13 (-0.36, 0.62) 2018 - 2021: -4.33 (-8.03, -0.47)* |
| WESTERN | 2 | -1.25 (-2.52; 0.05) | 1992 - 1998: -3.62 (-5.58, -1.61)* 1998 - 2001: 4.21 (-7.98, 18.02) 2001 - 2021: -1.32 (-1.68, -0.97)* |  | 2 | -1.42 (-1.80; -1.05)* | 1992 - 2007: -2.11 (-2.29, -1.93)* 2007 - 2019: -0.04 (-0.38, 0.30) 2019 - 2021: -4.46 (-9.38, 0.73) |
| UE28 | 5 | -1.76 (-2.31; -1.21)* | 1992 - 1994: 1.37 (-1.97, 4.82) 1994 - 1998: -4.32 (-5.96, -2.66)* 1998 - 2002: 0.19 (-1.57, 1.99) 2002 - 2013: -2.43 (-2.72, -2.14)* 2013 - 2016: 4.07 (-0.03, 8.33) 2016 - 2021: -4.34 (-5.23, -3.45)* |  | 3 | -2.33 (-2.62; -2.03)* | 1992 - 1996: -1.66 (-2.71, -0.60)* 1996 - 2010: -3.12 (-3.32, -2.93)* 2010 - 2016: 0.52 (-0.43, 1.48) 2016 - 2021: -3.97 (-4.95, -2.98)* |

AAPC: Anual Average percentage change. JP: Joinpoint. APC: Annual Percentage Change and 95% confidence interval. * = p<0.05

Western countries: green, Southern countries: red, Northern countries: blue, Central and Eastern countries: yellow.
