# Supplementary material for: Incidence and mortality of nonmelanoma skin cancer in Europe: current trends and challenges
Source: Clin Transl Oncol. 2025 Jul 11;28(1):302–19. doi: 10.1007/s12094-025-03985-z (PMC12790528; doi:10.1007/s12094-025-03985-z)
Supplement: Supplementary file 9 — Supplementary file9 (DOCX 21 KB) [file 12094_2025_3985_MOESM9_ESM.docx]

**Supplementary table 5.** Results of Joinpoint Analysis for SCC Mortality by Sex in 45-74 years old in European Countries (1992–2021).

| **Location** | **MEN** | | |  | **WOMEN** | | |
| --- | --- | --- | --- | --- | --- | --- | --- |
|  | **JP** | **AAPC 1992-2021** | **APC** |  | **JP** | **AAPC 1992-2021** | **APC** |
| Austria | 1 | -0.82 (-1.24; -0.40)* | 1992 - 2001: -5.54 (-6.68, -4.40)* 2001 - 2021: 1.38 (1.04, 1.73)* |  | 1 | -0.70 (-1.03; -0.37)* | 1992 - 2003: -4.31 (-5.01, -3.62)* 2003 - 2021: 1.57 (1.22, 1.92)* |
| Belgium | 3 | -0.47 (-1.21; 0.27) | 1992 - 1998: -3.59 (-5.52, -1.62)* 1998 - 2005: 4.89 (2.87, 6.94)* 2005 - 2016: -0.24 (-1.04, 0.56) 2016 - 2021: -4.40 (-6.52, -2.23)* |  | 1 | -0.40 (-0.83; 0.03) | 1992 - 2000: -1.72 (-3.10, -0.33)* 2000 - 2021: 0.11 (-0.21, 0.43) |
| Bulgaria | 1 | -0.24 (-1.11; 0.63) | 1992 - 2016: 0.89 (0.44, 1.35)* 2016 - 2021: -5.54 (-10.04, -0.81)* |  | 0 | -1.34 (-1.68; -1.00)* | 1992 - 2021: -1.34 (-1.68, -1.00)* |
| Croatia | 3 | -1.68 (-2.93; -0.42)* | 1992 - 2011: -1.66 (-1.94, -1.38)* 2011 - 2015: 5.72 (0.77, 10.92)* 2015 - 2018: -12.64 (-21.00, -3.39)* 2018 - 2021: 0.28 (-4.82, 5.65) |  | 2 | -3.79 (-5.55; -2.00)* | 1992 - 2012: -3.65 (-4.03, -3.27)* 2012 - 2015: 5.87 (-11.26, 26.30) 2015 - 2021: -8.72 (-11.45, -5.91)* |
| Cyprus | 4 | -1.02 (-1.39; -0.66)* | 1992 - 1998: 0.54 (-0.07, 1.16) 1998 - 2009: -1.85 (-2.11, -1.59)* 2009 - 2016: -0.47 (-0.97, 0.04) 2016 - 2019: 1.65 (-1.08, 4.44) 2019 - 2021: -6.84 (-9.36, -4.25)* |  | 4 | -1.77 (-2.08; -1.46)* | 1992 - 1998: -0.64 (-1.14, -0.14)* 1998 - 2003: -2.13 (-3.03, -1.22)* 2003 - 2007: -3.88 (-5.28, -2.45)* 2007 - 2019: -1.10 (-1.29, -0.92)* 2019 - 2021: -3.90 (-6.42, -1.31)* |
| Czechia | 1 | -2.12 (-2.79; -1.44)* | 1992 - 2016: -1.46 (-1.80, -1.11)* 2016 - 2021: -5.23 (-8.80, -1.52)* |  | 4 | -3.67 (-6.18; -1.09)* | 1992 - 2006: -2.53 (-3.19, -1.86)* 2006 - 2012: -6.82 (-10.40, -3.10)* 2012 - 2015: 12.92 (-5.19, 34.49) 2015 - 2018: -17.36 (-30.20, -2.15)* 2018 - 2021: -3.08 (-12.29, 7.09) |
| Denmark | 1 | 0.21 (-0.40; 0.82) | 1992 - 1995: 3.70 (-2.24, 10.01) 1995 - 2021: -0.19 (-0.38, 0.01) |  | 2 | -0.88 (-1.33; -0.42)* | 1992 - 1994: 3.23 (-3.06, 9.93) 1994 - 2009: -1.97 (-2.27, -1.68)* 2009 - 2021: -0.17 (-0.55, 0.20) |
| Estonia | 2 | -0.63 (-1.32; 0.06) | 1992 - 1999: 3.09 (1.74, 4.46)* 1999 - 2003: -3.79 (-8.11, 0.74) 2003 - 2021: -1.34 (-1.65, -1.02)* |  | 3 | -2.37 (-3.49; -1.25)* | 1992 - 1997: -0.62 (-2.46, 1.24) 1997 - 2000: 5.08 (-2.76, 13.55) 2000 - 2003: -8.52 (-15.71, -0.73)* 2003 - 2021: -2.99 (-3.31, -2.68)* |
| Finland | 2 | -0.37 (-1.16; 0.42) | 1992 - 1999: -2.57 (-3.60, -1.53)* 1999 - 2002: 2.91 (-4.65, 11.06) 2002 - 2021: -0.06 (-0.26, 0.14) |  | 2 | -0.93 (-1.33; -0.53)* | 1992 - 1996: -4.32 (-6.54, -2.05)* 1996 - 2008: -1.09 (-1.61, -0.57)* 2008 - 2021: 0.28 (-0.10, 0.67) |
| France | 4 | 0.24 (-0.76; 1.25) | 1992 - 1998: -1.17 (-2.61, 0.28) 1998 - 2001: 32.62 (23.31, 42.63)* 2001 - 2009: -0.53 (-1.26, 0.20) 2009 - 2012: -20.56 (-25.48, -15.32)* 2012 - 2021: 0.31 (-0.34, 0.95) |  | 0 | -0.19 (-0.36; -0.02)* | 1992 - 2021: -0.19 (-0.36, -0.02)* |
| Germany | 3 | 0.15 (-1.00; 1.32) | 1992 - 1997: -4.90 (-7.38, -2.35)* 1997 - 2004: 3.50 (1.61, 5.43)* 2004 - 2007: -2.94 (-12.19, 7.29) 2007 - 2021: 1.03 (0.58, 1.49)* |  | 4 | -0.38 (-0.97; 0.21) | 1992 - 1999: -3.72 (-4.53, -2.89)* 1999 - 2004: 2.16 (0.03, 4.33)* 2004 - 2009: -2.57 (-4.54, -0.56)* 2009 - 2016: 3.23 (2.15, 4.32)* 2016 - 2021: -0.89 (-2.20, 0.43) |
| Greece | 2 | 1.09 (0.30; 1.89)* | 1992 - 2011: 1.39 (1.05, 1.72)* 2011 - 2015: -3.43 (-8.33, 1.74) 2015 - 2021: 3.25 (1.50, 5.04)* |  | 2 | -0.38 (-0.66; -0.09)* | 1992 - 1998: -2.86 (-3.70, -2.01)* 1998 - 2005: -1.45 (-2.32, -0.57)* 2005 - 2021: 1.05 (0.85, 1.25)* |
| Hungary | 4 | -1.65 (-3.00; -0.27)* | 1992 - 1996: 2.40 (-0.25, 5.13) 1996 - 2013: -1.65 (-1.97, -1.33)* 2013 - 2016: 4.98 (-3.37, 14.05) 2016 - 2019: -15.14 (-22.56, -7.00)* 2019 - 2021: 2.71 (-6.82, 13.22) |  | 3 | -2.58 (-4.53; -0.59)* | 1992 - 1998: 0.39 (-2.21, 3.05) 1998 - 2012: -3.64 (-4.50, -2.77)* 2012 - 2015: 6.32 (-11.85, 28.24) 2015 - 2021: -7.16 (-10.07, -4.17)* |
| Ireland | 2 | -1.68 (-2.41; -0.95)* | 1992 - 1996: -4.26 (-7.31, -1.11)* 1996 - 2019: -0.50 (-0.73, -0.27)* 2019 - 2021: -9.62 (-17.43, -1.06)* |  | 2 | -2.07 (-2.47; -1.67)* | 1992 - 2006: -2.47 (-2.73, -2.20)* 2006 - 2019: -0.68 (-1.02, -0.35)* 2019 - 2021: -8.00 (-12.96, -2.76)* |
| Italy | 3 | -0.29 (-1.17; 0.60) | 1992 - 1994: 5.50 (-5.19, 17.39) 1994 - 2007: -3.20 (-3.81, -2.58)* 2007 - 2014: 4.35 (2.50, 6.22)* 2014 - 2021: -0.94 (-2.20, 0.34) |  | 3 | -0.72 (-1.69; 0.26) | 1992 - 1996: 1.35 (-2.10, 4.92) 1996 - 2010: -3.21 (-3.80, -2.62)* 2010 - 2014: 7.39 (1.26, 13.89)* 2014 - 2021: -1.30 (-2.74, 0.16) |
| Latvia | 1 | -0.14 (-0.45; 0.18) | 1992 - 2010: 1.09 (0.76, 1.42)* 2010 - 2021: -2.11 (-2.78, -1.43)* |  | 3 | -0.99 (-1.77; -0.20)* | 1992 - 1996: 0.34 (-1.99, 2.72) 1996 - 1999: 6.32 (-0.88, 14.05) 1999 - 2009: -1.52 (-2.15, -0.89)* 2009 - 2021: -2.73 (-3.21, -2.26)* |
| Lithuania | 3 | 0.63 (-0.20; 1.47) | 1992 - 1998: 3.90 (2.13, 5.69)* 1998 - 2002: -2.35 (-6.63, 2.14) 2002 - 2007: 3.17 (0.31, 6.12)* 2007 - 2021: -0.77 (-1.19, -0.35)* |  | 3 | -1.09 (-1.92; -0.25)* | 1992 - 2000: 2.50 (1.63, 3.37)* 2000 - 2003: -5.67 (-12.50, 1.71) 2003 - 2010: 0.81 (-0.51, 2.15) 2010 - 2021: -3.54 (-4.09, -2.98)* |
| Luxembourg | 4 | -0.92 (-1.75; -0.08)* | 1992 - 1999: -2.41 (-3.40, -1.42)* 1999 - 2002: 2.31 (-4.68, 9.82) 2002 - 2012: -1.01 (-1.63, -0.38)* 2012 - 2017: 2.36 (0.32, 4.44)* 2017 - 2021: -4.42 (-6.18, -2.64)* |  | 4 | -1.36 (-2.06; -0.65)* | 1992 - 1999: -3.40 (-4.34, -2.44)* 1999 - 2003: 1.86 (-1.89, 5.76) 2003 - 2010: -2.42 (-3.66, -1.17)* 2010 - 2016: 3.77 (2.13, 5.43)* 2016 - 2021: -5.43 (-6.84, -4.00)* |
| Malta | 3 | -0.31 (-1.17; 0.55) | 1992 - 2004: 0.24 (-0.34, 0.82) 2004 - 2015: -1.34 (-2.00, -0.68)* 2015 - 2018: 4.68 (-2.81, 12.74) 2018 - 2021: -3.54 (-6.98, 0.02) |  | 2 | -1.10 (-1.81; -0.38)* | 1992 - 1997: -3.49 (-5.69, -1.23)* 1997 - 2019: -1.18 (-1.43, -0.93)* 2019 - 2021: 6.08 (-3.11, 16.15) |
| Netherlands | 1 | -1.02 (-1.53; -0.52)* | 1992 - 2017: -0.60 (-0.85, -0.36)* 2017 - 2021: -3.61 (-7.01, -0.09)* |  | 2 | -0.57 (-1.18; 0.04) | 1992 - 2009: -1.41 (-1.72, -1.11)* 2009 - 2013: 4.31 (0.02, 8.79)* 2013 - 2021: -1.15 (-1.97, -0.32)* |
| Poland | 3 | -8.46 (-11.02; -5.83)* | 1992 - 2005: -1.25 (-1.84, -0.67)* 2005 - 2014: -13.39 (-14.85, -11.90)* 2014 - 2017: -25.76 (-42.49, -4.16)* 2017 - 2021: -5.17 (-14.07, 4.66) |  | 5 | -8.84 (-11.06; -6.57)* | 1992 - 1999: -0.43 (-1.48, 0.64) 1999 - 2003: -6.15 (-9.94, -2.19)* 2003 - 2006: -1.40 (-9.70, 7.68) 2006 - 2014: -15.58 (-16.95, -14.18)* 2014 - 2017: -24.11 (-38.56, -6.26)* 2017 - 2021: -4.31 (-11.54, 3.50) |
| Portugal | 1 | -0.69 (-1.09; -0.28)* | 1992 - 1999: -3.20 (-4.72, -1.66)* 1999 - 2021: 0.13 (-0.14, 0.40) |  | 4 | -1.69 (-2.97; -0.40)* | 1992 - 2003: -2.58 (-3.12, -2.04)* 2003 - 2006: -6.80 (-14.89, 2.06) 2006 - 2009: 6.52 (-2.47, 16.34) 2009 - 2017: -0.10 (-1.19, 1.01) 2017 - 2021: -4.35 (-6.88, -1.76)* |
| Romania | 3 | -0.51 (-1.44; 0.43) | 1992 - 1995: 3.18 (-0.90, 7.44) 1995 - 2013: -1.64 (-1.91, -1.37)* 2013 - 2016: 5.18 (-3.08, 14.15) 2016 - 2021: -1.90 (-3.61, -0.16)* |  | 2 | -2.66 (-4.48; -0.80)* | 1992 - 2012: -3.14 (-3.56, -2.71)* 2012 - 2015: 6.78 (-10.92, 28.00) 2015 - 2021: -5.53 (-8.35, -2.62)* |
| Slovakia |  | -1.38 (-1.47; -1.29)* |  |  | 2 | -2.21 (-2.42; -1.99)* | 1992 - 1997: -1.79 (-2.67, -0.91)* 1997 - 2013: -2.98 (-3.16, -2.80)* 2013 - 2021: -0.92 (-1.42, -0.41)* |
| Slovenia | 1 | -3.33 (-5.19; -1.43)* | 1992 - 2002: -1.60 (-1.83, -1.38)* 2002 - 2021: -1.26 (-1.34, -1.17)* |  | 3 | -5.63 (-7.52; -3.70)* | 1992 - 2005: -3.53 (-4.22, -2.84)* 2005 - 2012: -6.58 (-9.19, -3.89)* 2012 - 2015: 8.29 (-9.84, 30.07) 2015 - 2021: -15.01 (-17.87, -12.04)* |
| Spain | 4 | -0.90 (-1.45; -0.35)* | 1992 - 2003: -1.80 (-2.56, -1.03)* 2003 - 2013: -3.62 (-4.66, -2.58)* 2013 - 2016: 7.98 (-3.82, 21.23) 2016 - 2019: -19.50 (-29.29, -8.36)* 2019 - 2021: 0.37 (-12.95, 15.73) |  | 2 | -1.47 (-1.89; -1.06)* | 1992 - 2004: -2.82 (-3.31, -2.32)* 2004 - 2013: 0.64 (-0.32, 1.62) 2013 - 2021: -1.79 (-2.70, -0.88)* |
| Sweden | 2 | -1.03 (-2.60; 0.56) | 1992 - 2000: -2.64 (-3.86, -1.42)* 2000 - 2015: -0.81 (-1.34, -0.27)* 2015 - 2021: 1.23 (-0.60, 3.10) |  | 0 | 0.62 (0.18; 1.06)* | 1992 - 2021: 0.62 (0.18, 1.06)* |
| United Kingdom | 6 | -0.35 (-1.82; 1.14) | 1992 - 1994: 0.63 (-7.87, 9.92) 1994 - 1999: -12.12 (-15.00, -9.13)* 1999 - 2002: 15.26 (3.82, 27.96)* 2002 - 2010: 1.12 (-0.13, 2.39) 2010 - 2013: -11.42 (-19.16, -2.94)* 2013 - 2018: 10.25 (7.34, 13.24)* 2018 - 2021: -9.74 (-13.40, -5.94)* |  | 3 | -0.61 (-2.05; 0.86) | 1992 - 1996: -5.01 (-9.02, -0.82)* 1996 - 2013: -0.70 (-1.26, -0.13)* 2013 - 2016: 16.90 (2.79, 32.96)* 2016 - 2021: -6.20 (-8.64, -3.70)* |
| CENTRAL/  EASTERN | 3 | -2.80 (-3.17; -2.42)* | 1992 - 1997: -4.45 (-7.66, -1.12)* 1997 - 2013: -0.38 (-1.04, 0.28) 2013 - 2016: 21.72 (6.82, 38.70)* 2016 - 2021: -7.73 (-10.10, -5.29)* |  | 1 | -3.82 (-4.35; -3.28)* | 1992 - 1998: -0.46 (-2.80, 1.94) 1998 - 2021: -4.68 (-5.06, -4.30)* |
| NORTHERN | 1 | -0.42 (-1.38; 0.55) | 1992 - 2003: -0.91 (-1.70, -0.11)* 2003 - 2021: -3.94 (-4.35, -3.52)* |  | 2 | -0.64 (-1.27; -0.01)* | 1992 - 2012: -1.25 (-1.48, -1.01)* 2012 - 2016: 7.26 (2.89, 11.82)* 2016 - 2021: -4.24 (-5.89, -2.57)* |
| SOUTHERN | 3 | -0.51 (-1.02; -0.00)* | 1992 - 1997: -3.17 (-5.27, -1.02)* 1997 - 2013: -0.35 (-0.76, 0.07) 2013 - 2016: 14.11 (4.79, 24.27)* 2016 - 2021: -5.85 (-7.48, -4.20)* |  | 2 | -1.33 (-1.72; -0.94)* | 1992 - 2008: -2.44 (-2.70, -2.17)* 2008 - 2015: 1.67 (0.42, 2.93)* 2015 - 2021: -1.80 (-2.96, -0.63)* |
| WESTERN | 4 | 0.06 (-0.97; 1.10) | 1992 - 1994: 3.71 (-1.37, 9.06) 1994 - 1999: -3.64 (-5.12, -2.13)* 1999 - 2007: -1.01 (-1.68, -0.34)* 2007 - 2018: 0.81 (0.44, 1.19)* 2018 - 2021: -1.46 (-3.67, 0.79) |  | 4 | -0.45 (-1.04; 0.15) | 1992 - 1999: -2.83 (-3.47, -2.18)* 1999 - 2002: 2.91 (-2.18, 8.27) 2002 - 2008: -1.55 (-2.64, -0.45)* 2008 - 2017: 1.49 (0.97, 2.02)* 2017 - 2021: -1.35 (-2.75, 0.06) |
| UE28 | 4 | -0.91 (-1.48; -0.34)* | 1992 - 1998: -2.50 (-4.08, -0.88)* 1998 - 2002: 12.52 (7.75, 17.51)* 2002 - 2009: -1.03 (-2.26, 0.22) 2009 - 2012: -8.76 (-15.53, -1.45)* 2012 - 2021: 0.50 (-0.23, 1.24) |  | 3 | -1.65 (-1.96; -1.34)* | 1992 - 2002: -1.80 (-2.11, -1.50)* 2002 - 2011: -2.57 (-3.02, -2.12)* 2011 - 2016: 1.94 (0.59, 3.31)* 2016 - 2021: -3.21 (-4.11, -2.30)* |

AAPC: Anual Average percentage change. JP: Joinpoint. APC: Annual Percentage Change and 95% confidence interval. * = p<0.05

Western countries: green, Southern countries: red, Northern countries: blue, Central and Eastern countries: yellow.
