# Supplementary material for: Incidence and mortality of nonmelanoma skin cancer in Europe: current trends and challenges
Source: Clin Transl Oncol. 2025 Jul 11;28(1):302–19. doi: 10.1007/s12094-025-03985-z (PMC12790528; doi:10.1007/s12094-025-03985-z)
Supplement: Supplementary file 10 — Supplementary file10 (DOCX 23 KB) [file 12094_2025_3985_MOESM10_ESM.docx]

**Supplementary table 6.** Results of Joinpoint Analysis for SCC Mortality by Sex in > 74 years old in European Countries (1992–2021).

| **Location** | **MEN** | | |  | **WOMEN** | | |
| --- | --- | --- | --- | --- | --- | --- | --- |
|  | **JP** | **AAPC 1992-2021** | **APC** |  | **JP** | **AAPC 1992-2021** | **APC** |
| Austria | 1 | 0.86 (0.46; 1.27)* | 1992 - 1999: -3.80 (-5.35, -2.21)* 1999 - 2021: 2.40 (2.18, 2.61)* |  | 1 | 0.45 (-0.09; 0.98) | 1992 - 2000: -4.19 (-5.90, -2.44)* 2000 - 2021: 2.27 (1.91, 2.63)* |
| Belgium | 4 | -0.21 (-1.18; 0.78) | 1992 - 1999: -1.93 (-3.31, -0.53)* 1999 - 2003: 4.45 (-0.43, 9.58) 2003 - 2014: -0.22 (-0.83, 0.40) 2014 - 2017: 2.88 (-3.49, 9.67) 2017 - 2021: -3.89 (-5.78, -1.96)* |  | 2 | -0.47 (-1.25; 0.31) | 1992 - 1995: -3.41 (-8.66, 2.14) 1995 - 2019: 0.37 (0.17, 0.58)* 2019 - 2021: -5.96 (-13.42, 2.14) |
| Bulgaria | 2 | -0.06 (-1.38; 1.29) | 1992 - 2013: 0.46 (-0.10, 1.01) 2013 - 2016: 6.71 (-5.73, 20.80) 2016 - 2021: -5.96 (-8.36, -3.50)* |  | 3 | -2.22 (-4.11; -0.29)* | 1992 - 2013: -2.04 (-2.60, -1.48)* 2013 - 2016: 7.66 (-5.25, 22.32) 2016 - 2019: -14.25 (-24.51, -2.59)* 2019 - 2021: 1.15 (-10.42, 14.21) |
| Croatia | 4 | -2.20 (-3.38; -0.99)* | 1992 - 1995: -8.50 (-12.91, -3.87)* 1995 - 2011: -1.53 (-2.01, -1.06)* 2011 - 2015: 6.24 (1.20, 11.52)* 2015 - 2018: -10.82 (-18.12, -2.87)* 2018 - 2021: -0.94 (-4.97, 3.26) |  | 4 | -2.15 (-3.62; -0.66)* | 1992 - 1997: -7.60 (-9.95, -5.20)* 1997 - 2012: -1.91 (-2.47, -1.36)* 2012 - 2015: 14.61 (3.37, 27.08)* 2015 - 2018: -11.94 (-20.02, -3.04)* 2018 - 2021: 0.91 (-3.93, 6.00) |
| Cyprus | 4 | -3.07 (-3.89; -2.24)* | 1992 - 1997: -9.01 (-10.56, -7.43)* 1997 - 2002: -0.49 (-2.75, 1.82) 2002 - 2005: -9.72 (-15.70, -3.32)* 2005 - 2015: 1.87 (1.27, 2.47)* 2015 - 2021: -4.64 (-5.74, -3.53)* |  | 4 | -2.49 (-3.64; -1.33)* | 1992 - 1997: -7.23 (-9.30, -5.10)* 1997 - 2002: -1.45 (-4.29, 1.47) 2002 - 2005: -8.49 (-17.12, 1.05) 2005 - 2012: 0.99 (-0.73, 2.74) 2012 - 2021: -0.96 (-1.41, -0.50)* |
| Czechia | 4 | -1.58 (-3.12; -0.02)* | 1992 - 1995: -10.61 (-17.53, -3.11)* 1995 - 2004: 3.23 (1.58, 4.89)* 2004 - 2012: -3.80 (-5.56, -2.00)* 2012 - 2015: 7.90 (-4.38, 21.77) 2015 - 2021: -5.36 (-7.07, -3.61)* |  | 5 | -2.98 (-4.64; -1.29)* | 1992 - 1998: -6.06 (-7.82, -4.26)* 1998 - 2003: 2.75 (-0.88, 6.50) 2003 - 2012: -5.47 (-6.69, -4.23)* 2012 - 2015: 10.95 (-0.84, 24.13) 2015 - 2018: -13.05 (-21.80, -3.31)* 2018 - 2021: -0.79 (-6.36, 5.11) |
| Denmark | 1 | 1.91 (1.40; 2.42)* | 1992 - 2001: 3.35 (1.79, 4.94)* 2001 - 2021: 1.27 (0.94, 1.60)* |  | 0 | 0.91 (0.74; 1.08)* | 1992 - 2021: 0.91 (0.74, 1.08)* |
| Estonia | 3 | 0.92 (-0.14; 1.99) | 1992 - 1999: 4.10 (2.37, 5.87)* 1999 - 2003: -3.09 (-8.21, 2.31) 2003 - 2007: 3.55 (-1.67, 9.05) 2007 - 2021: -0.21 (-0.60, 0.19) |  | 3 | -0.36 (-1.18; 0.46) | 1992 - 1999: 3.06 (1.62, 4.52)* 1999 - 2006: -3.90 (-5.48, -2.29)* 2006 - 2010: 3.57 (-1.35, 8.73) 2010 - 2021: -1.61 (-2.16, -1.05)* |
| Finland | 2 | 0.32 (-0.41; 1.06) | 1992 - 2013: 0.48 (0.14, 0.82)* 2013 - 2017: 3.10 (-1.57, 7.98) 2017 - 2021: -3.17 (-5.73, -0.54)* |  | 3 | -0.14 (-0.93; 0.66) | 1992 - 2002: -0.43 (-1.21, 0.35) 2002 - 2006: -3.41 (-7.98, 1.38) 2006 - 2018: 1.68 (1.10, 2.27)* 2018 - 2021: -1.89 (-5.43, 1.80) |
| France | 5 | 0.83 (0.32; 1.35)* | 1992 - 1999: 1.11 (0.47, 1.76)* 1999 - 2002: 9.24 (5.19, 13.43)* 2002 - 2010: -0.28 (-0.71, 0.15) 2010 - 2013: -3.22 (-6.01, -0.35)* 2013 - 2018: 1.99 (1.15, 2.84)* 2018 - 2021: -2.62 (-3.76, -1.46)* |  | 4 | 0.07 (-0.79; 0.94) | 1992 - 1999: -0.36 (-1.28, 0.57) 1999 - 2002: 6.11 (-0.31, 12.95) 2002 - 2009: -1.08 (-2.05, -0.10)* 2009 - 2012: 2.46 (-3.05, 8.29) 2012 - 2021: -1.42 (-1.86, -0.97)* |
| Germany | 3 | 1.04 (0.48; 1.61)* | 1992 - 1998: -4.57 (-6.20, -2.91)* 1998 - 2011: 1.70 (1.16, 2.23)* 2011 - 2016: 5.69 (3.47, 7.95)* 2016 - 2021: 1.75 (0.52, 3.00)* |  | 3 | 0.19 (-0.18; 0.56) | 1992 - 1998: -6.20 (-7.09, -5.31)* 1998 - 2010: 1.22 (0.85, 1.60)* 2010 - 2019: 3.69 (3.20, 4.18)* 2019 - 2021: -1.67 (-5.37, 2.18) |
| Greece | 3 | 0.36 (-0.15; 0.88) | 1992 - 2003: 1.12 (0.54, 1.71)* 2003 - 2013: -1.78 (-2.42, -1.15)* 2013 - 2019: 3.39 (2.07, 4.73)* 2019 - 2021: -1.93 (-6.95, 3.37) |  | 2 | -1.06 (-1.54; -0.58)* | 1992 - 2004: 1.42 (0.87, 1.98)* 2004 - 2011: -4.37 (-5.98, -2.73)* 2011 - 2021: -1.64 (-2.25, -1.02)* |
| Hungary | 2 | -2.05 (-2.80; -1.29)* | 1992 - 2010: -1.61 (-2.10, -1.12)* 2010 - 2016: 2.88 (0.04, 5.81)* 2016 - 2021: -9.13 (-11.59, -6.59)* |  | 3 | -2.71 (-3.63; -1.78)* | 1992 - 1998: -0.33 (-2.70, 2.10) 1998 - 2009: -5.35 (-6.40, -4.29)* 2009 - 2015: 3.43 (0.30, 6.67)* 2015 - 2021: -6.04 (-8.04, -4.00)* |
| Ireland | 3 | -0.93 (-1.75; -0.11)* | 1992 - 1998: -1.83 (-4.01, 0.40) 1998 - 2005: 1.58 (-0.35, 3.55) 2005 - 2019: -0.60 (-1.06, -0.14)* 2019 - 2021: -8.87 (-15.76, -1.40)* |  | 2 | -0.73 (-1.30; -0.15)* | 1992 - 2004: -1.49 (-2.22, -0.75)* 2004 - 2018: 1.23 (0.70, 1.76)* 2018 - 2021: -6.56 (-10.69, -2.23)* |
| Italy | 4 | 0.92 (0.15; 1.70)* | 1992 - 1997: 3.55 (0.92, 6.25)* 1997 - 2007: -2.24 (-3.09, -1.37)* 2007 - 2012: 5.67 (2.93, 8.49)* 2012 - 2018: 2.39 (0.87, 3.93)* 2018 - 2021: -3.26 (-6.16, -0.27)* |  | 4 | -0.19 (-1.18; 0.82) | 1992 - 2000: 0.63 (-0.55, 1.82) 2000 - 2006: -5.73 (-7.74, -3.67)* 2006 - 2010: 0.83 (-4.00, 5.90) 2010 - 2014: 5.98 (1.51, 10.64)* 2014 - 2021: -0.22 (-1.23, 0.79) |
| Latvia | 1 | 1.27 (0.78; 1.77)* | 1992 - 2017: 1.95 (1.68, 2.23)* 2017 - 2021: -2.87 (-6.07, 0.44) |  | 1 | 0.13 (-0.42; 0.68) | 1992 - 2012: 1.42 (0.89, 1.96)* 2012 - 2021: -2.69 (-4.09, -1.26)* |
| Lithuania | 4 | 1.58 (0.63; 2.53)* | 1992 - 1996: 0.78 (-1.65, 3.26) 1996 - 1999: 7.70 (0.16, 15.81)* 1999 - 2003: -2.53 (-5.96, 1.03) 2003 - 2010: 4.39 (3.14, 5.65)* 2010 - 2021: 0.02 (-0.38, 0.41) |  | 3 | -0.03 (-1.12; 1.07) | 1992 - 1999: 3.75 (2.21, 5.30)* 1999 - 2002: -4.91 (-13.97, 5.09) 2002 - 2016: 0.48 (-0.02, 0.99) 2016 - 2021: -3.57 (-5.40, -1.71)* |
| Luxembourg | 2 | 0.56 (0.01; 1.12)* | 1992 - 2013: 0.60 (0.31, 0.88)* 2013 - 2018: 3.77 (1.27, 6.33)* 2018 - 2021: -4.82 (-7.95, -1.58)* |  | 4 | -0.65 (-1.24; -0.05)* | 1992 - 1999: -2.98 (-3.91, -2.05)* 1999 - 2003: 1.45 (-1.83, 4.85) 2003 - 2010: -1.10 (-2.12, -0.06)* 2010 - 2016: 3.33 (2.10, 4.58)* 2016 - 2021: -3.04 (-4.09, -1.97)* |
| Malta | 4 | -0.45 (-1.11; 0.23) | 1992 - 1997: -2.68 (-4.87, -0.43)* 1997 - 2003: 3.23 (1.26, 5.23)* 2003 - 2011: -1.57 (-2.53, -0.61)* 2011 - 2018: 1.89 (0.83, 2.96)* 2018 - 2021: -6.10 (-8.64, -3.49)* |  | 2 | -0.40 (-0.97; 0.19) | 1992 - 2008: -1.16 (-1.60, -0.72)* 2008 - 2013: 2.21 (-0.79, 5.31) 2013 - 2021: -0.47 (-1.33, 0.39) |
| Netherlands | 1 | 0.84 (0.51; 1.18)* | 1992 - 2008: -0.01 (-0.50, 0.49) 2008 - 2021: 1.90 (1.40, 2.41)* |  | 2 | 0.32 (-0.19; 0.84) | 1992 - 2007: -1.14 (-1.62, -0.66)* 2007 - 2017: 2.99 (2.07, 3.92)* 2017 - 2021: -0.72 (-3.36, 1.99) |
| Poland | 3 | -7.58 (-9.58; -5.53)* | 1992 - 1995: -7.96 (-16.92, 1.97) 1995 - 2006: -0.03 (-1.53, 1.49) 2006 - 2018: -16.00 (-17.54, -14.44)* 2018 - 2021: 2.02 (-14.75, 22.08) |  | 4 | -9.24 (-11.91; -6.50)* | 1992 - 2007: -3.53 (-3.97, -3.09)* 2007 - 2011: -21.24 (-26.62, -15.47)* 2011 - 2014: -8.40 (-22.86, 8.78) 2014 - 2017: -28.78 (-42.49, -11.80)* 2017 - 2021: -0.92 (-8.90, 7.75) |
| Portugal | 1 | -0.61 (-1.07; -0.16)* | 1992 - 2005: -2.16 (-3.06, -1.25)* 2005 - 2021: 0.66 (0.22, 1.10)* |  | 3 | -0.98 (-2.48; 0.55) | 1992 - 2002: -0.87 (-1.79, 0.07) 2002 - 2005: -11.03 (-20.35, -0.61)* 2005 - 2008: 5.87 (-4.82, 17.76) 2008 - 2021: -0.14 (-0.58, 0.30) |
| Romania | 2 | -1.57 (-2.47; -0.67)* | 1992 - 2011: -1.67 (-2.11, -1.23)* 2011 - 2015: 6.17 (-0.15, 12.89) 2015 - 2021: -6.12 (-7.80, -4.41)* |  | 2 | -3.24 (-4.83; -1.63)* | 1992 - 2012: -3.18 (-3.67, -2.69)* 2012 - 2015: 4.03 (-11.25, 21.93) 2015 - 2021: -6.87 (-9.07, -4.61)* |
| Slovakia | 2 | -0.82 (-1.07; -0.57)* | 1992 - 2004: -2.49 (-2.81, -2.16)* 2004 - 2014: 1.59 (1.10, 2.09)* 2014 - 2021: -1.33 (-1.99, -0.68)* |  | 4 | -1.46 (-1.83; -1.08)* | 1992 - 1998: -2.56 (-3.21, -1.90)* 1998 - 2002: -4.34 (-6.24, -2.41)* 2002 - 2008: -1.08 (-1.98, -0.18)* 2008 - 2015: 0.87 (0.22, 1.52)* 2015 - 2021: -1.46 (-2.04, -0.88)* |
| Slovenia | 1 | 0.09 (-0.64; 0.83) | 1992 - 2016: 1.17 (0.70, 1.63)* 2016 - 2021: -4.89 (-8.53, -1.11)* |  | 3 | -2.55 (-3.89; -1.18)* | 1992 - 2013: -1.92 (-2.18, -1.65)* 2013 - 2016: 6.34 (-2.77, 16.32) 2016 - 2019: -15.10 (-22.35, -7.16)* 2019 - 2021: -1.73 (-10.79, 8.25) |
| Spain | 1 | -0.73 (-1.06; -0.40)* | 1992 - 2000: -2.92 (-4.03, -1.80)* 2000 - 2021: 0.11 (-0.09, 0.32) |  | 1 | -2.12 (-2.37; -1.87)* | 1992 - 2005: -3.86 (-4.31, -3.40)* 2005 - 2021: -0.69 (-0.99, -0.39)* |
| Sweden | 6 | -0.00 (-2.86; 2.93) | 1992 - 1999: -6.89 (-9.29, -4.43)* 1999 - 2002: 15.63 (-3.44, 38.46) 2002 - 2005: -4.99 (-19.48, 12.11) 2005 - 2008: 11.19 (-4.50, 29.45) 2008 - 2013: -5.13 (-9.39, -0.67)* 2013 - 2018: 7.54 (3.14, 12.14)* 2018 - 2021: -6.50 (-12.10, -0.55)* |  | 0 | 1.88 (1.46; 2.30)* | 1992 - 2021: 1.88 (1.46, 2.30)* |
| United Kingdom | 2 | 1.02 (0.28; 1.76)* | 1992 - 2011: -0.31 (-0.82, 0.20) 2011 - 2017: 11.03 (8.20, 13.94)* 2017 - 2021: -6.61 (-9.60, -3.53)* |  | 2 | 0.95 (-0.06; 1.97) | 1992 - 2012: -0.17 (-0.61, 0.26) 2012 - 2016: 12.52 (5.29, 20.25)* 2016 - 2021: -3.23 (-5.71, -0.67)* |
| CENTRAL/  EASTERN | 4 | -3.21 (-4.24; -2.17)* | 1992 - 1995: -5.66 (-10.89, -0.12)* 1995 - 2006: -0.34 (-1.14, 0.46) 2006 - 2011: -7.56 (-10.57, -4.45)* 2011 - 2015: 1.10 (-3.77, 6.20) 2015 - 2021: -6.21 (-7.71, -4.69)* |  | 4 | -4.61 (-5.93; -3.28)* | 1992 - 2005: -2.78 (-3.20, -2.36)* 2005 - 2012: -8.52 (-9.90, -7.11)* 2012 - 2015: 1.59 (-7.40, 11.46) 2015 - 2018: -12.60 (-20.33, -4.12)* 2018 - 2021: -0.75 (-5.49, 4.22) |
| NORTHERN | 2 | 1.15 (0.15; 2.17)* | 1992 - 2013: 0.48 (0.15, 0.82)* 2013 - 2016: 16.02 (5.57, 27.51)* 2016 - 2021: -4.20 (-5.94, -2.42)* |  | 2 | 0.74 (0.16; 1.32)* | 1992 - 2010: -0.09 (-0.48, 0.30) 2010 - 2017: 5.40 (3.67, 7.16)* 2017 - 2021: -3.41 (-6.16, -0.58)* |
| SOUTHERN | 2 | -0.13 (-0.41; 0.16) | 1992 - 2006: -1.21 (-1.52, -0.89)* 2006 - 2018: 1.71 (1.36, 2.06)* 2018 - 2021: -2.33 (-4.38, -0.24)* |  | 3 | -1.19 (-1.60; -0.77)* | 1992 - 2001: -1.76 (-2.32, -1.19)* 2001 - 2006: -4.92 (-6.68, -3.12)* 2006 - 2016: 1.24 (0.74, 1.74)* 2016 - 2021: -1.14 (-2.19, -0.09)* |
| WESTERN | 4 | 0.87 (0.45; 1.29)* | 1992 - 1999: -1.43 (-1.99, -0.87)* 1999 - 2002: 6.73 (2.90, 10.70)* 2002 - 2012: 0.36 (0.08, 0.65)* 2012 - 2019: 2.46 (2.03, 2.89)* 2019 - 2021: -2.49 (-4.60, -0.33)* |  | 2 | 0.15 (-0.28; 0.57) | 1992 - 1998: -3.08 (-4.26, -1.88)* 1998 - 2019: 1.27 (1.10, 1.44)* 2019 - 2021: -1.73 (-6.54, 3.33) |
| UE28 | 5 | -0.03 (-0.33; 0.28) | 1992 - 1999: -1.47 (-1.80, -1.15)* 1999 - 2002: 2.42 (0.16, 4.74)* 2002 - 2009: -0.86 (-1.20, -0.51)* 2009 - 2013: 0.67 (-0.26, 1.61) 2013 - 2016: 4.98 (3.25, 6.74)* 2016 - 2021: -1.74 (-2.07, -1.41)* |  | 4 | -1.05 (-1.30; -0.79)* | 1992 - 1999: -2.02 (-2.32, -1.71)* 1999 - 2002: -0.27 (-2.46, 1.97) 2002 - 2010: -2.11 (-2.40, -1.82)* 2010 - 2016: 1.70 (1.25, 2.16)* 2016 - 2021: -1.69 (-2.10, -1.27)* |

AAPC: Anual Average percentage change. JP: Joinpoint. APC: Annual Percentage Change and 95% confidence interval. * = p<0.05

Western countries: green, Southern countries: red, Northern countries: blue, Central and Eastern countries: yellow.
